# Temporal Dynamics and Regulatory Control of Ginsenoside Accumulation During Leaf Senescence in *Panax ginseng*

**DOI:** 10.3390/plants15203209

**Published:** 2026-10-19

**Authors:** Heng Wei, Wenhao Jia, Junbo Rong, Mengyang Zhang, Peng Di, Jian Zhang, Hongmei Lin, Yingping Wang

**Affiliations:** 1 College of Chinese Medicinal Materials, Jilin Agricultural University, 2888 Xincheng Street, Jingyue Development Zone, Changchun 130118, China; weih002@163.com (H.W.); jiawenhao32@mails.jlau.edu.cn (W.J.); 20242146@mails.jlau.edu.cn (J.R.); 15567131068@163.com (M.Z.); di@jlau.edu.cn (P.D.); jian.zhang@ubc.ca (J.Z.); 2 Department of Biology, Okanagan Campus, University of British Columbia, Kelowna, BC V1V 1V7, Canada

**Keywords:** *Panax ginseng*, leaf senescence, ginsenosides, targeted metabolomics, transcriptome, generalized estimating equations

## Abstract

Ginsenosides, the principal bioactive triterpenoid saponins of *Panax ginseng* C.A. Meyer, accumulate abundantly in leaves. They form a large family of secondary metabolites comprising more than 300 structurally distinct compounds, yet how leaf senescence reshapes their accumulation remains unclear. We profiled 376 saponin metabolite features across four sampling stages spanning the natural senescence progression (T1–T3) and a frost-terminated stage (T4) by ultra-performance liquid chromatography–high-resolution mass spectrometry (UPLC-HRMS), modeled temporal effects across the natural senescence gradient (T1–T3) with generalized estimating equations (GEE), analyzed the frost-terminated stage T4 as a distinct endpoint, verified senescence histologically, and analyzed stage T1–T3 transcriptomes with replicate-based statistical testing. Leaf senescence globally reshaped the saponin metabolome, with 326 of the 376 saponin features (86.7%) showing significant temporal effects across the natural senescence gradient (T1–T3). The four classical aglycone classes—protopanaxadiol (PPD), protopanaxatriol (PPT), ocotillol (OT) and oleanolic acid (OA)—followed divergent accumulation programs across the natural senescence progression (T1–T3): sustained accumulation (PPT, OT and PPD) versus a U-shaped curve (OA). At the frost-terminated stage, T4, the classes diverged sharply—PPD-type abundance fell back to the T1 level (abundance-weighted class-level T4/T3 = 0.63), whereas the other classes rose further—a frost-associated remodeling pattern rather than a continuation of the senescence program. The frost-associated PPD decline was glycosylation-dependent—low-glycosylation compounds fell far more sharply than highly glycosylated ones (median of per-compound ratios T4/T3 0.51 vs. 1.04; one-sided permutation *p* = 1.2 × 10^−4^)—and coincided with T3 induction of β-glucosidase genes (exploratory and hypothesis-level, based on two qualified T3 replicates, and requiring validation with larger replicate numbers) and histologically verified vacuolar collapse, consistent with a deglycosylation-associated catabolic mechanism. Senescence signaling was consistent with differential hormone responses (salicylic acid activation, jasmonate and ethylene attenuation), broad suppression of Ca^2+^-signaling components and WRKY/NAC-centered transcriptional control, and joint analysis suggested a dichotomous saponin–gene correlation pattern. Collectively, these results establish a temporal atlas of ginsenoside accumulation during ginseng leaf senescence, delineate the class-specific coupling of saponin secondary metabolism to the senescence program, and offer a new perspective on ginsenosides as metabolites embedded in the physiological activities of the ginseng life cycle.

## 1. Introduction

**Figure 1:**
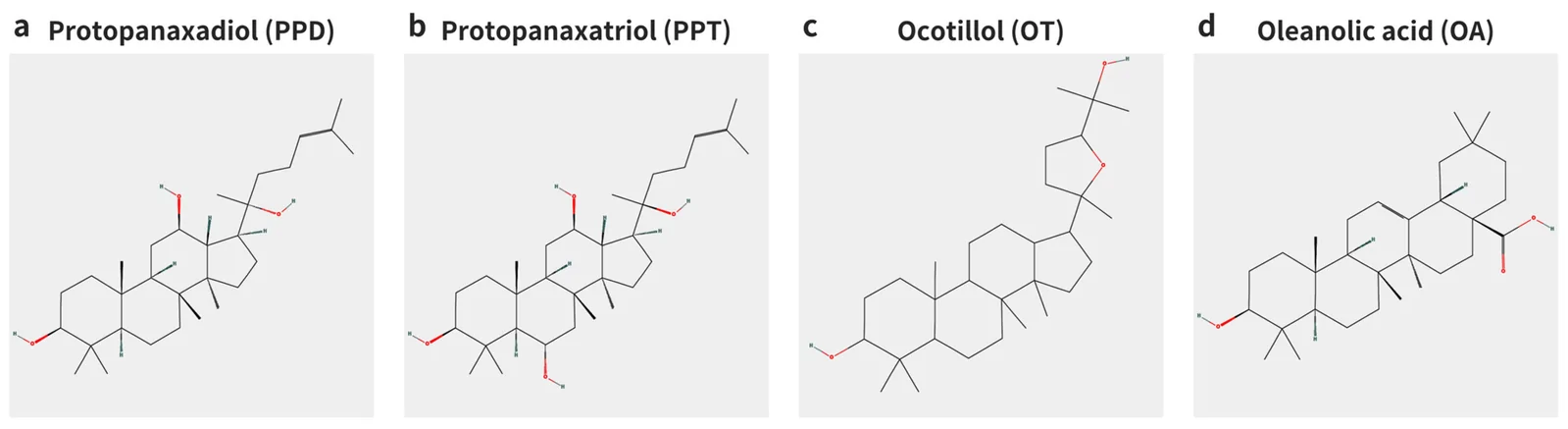
Chemical structures of the four classical ginsenoside aglycones: (**a**) protopanaxadiol (PPD); (**b**) protopanaxatriol (PPT); (**c**) ocotillol (OT); (**d**) oleanolic acid (OA). Two-dimensional structures were retrieved from PubChem (compound identifiers 9920281, 11468733, 3850493 and 10494, respectively).

Ginseng (*Panax ginseng* C.A. Meyer) is one of the most valued traditional medicinal plants, and its major active constituents, the ginsenosides, are triterpenoid saponins with broad pharmacological activities, including antitumor, immunomodulatory, neuroprotective and anti-aging effects [1,2]. According to their aglycone structures, ginsenosides are classified into protopanaxadiol (PPD), protopanaxatriol (PPT), ocotillol (OT) and oleanolic acid (OA) types [1,3] (chemical structures of the four aglycones are shown in Figure 1), and systematic surveys by Guo and colleagues have cataloged the remarkable chemical diversity of saponins across the genus *Panax*, including hundreds of potential new ginsenosides discovered by LC/MS-based strategies [4,5,6]. Their biosynthesis proceeds through the mevalonate (MVA) and methylerythritol phosphate (MEP) pathways, followed by squalene epoxidation and cyclization, cytochrome P450-mediated oxidation—including CYP716A47, which forms PPD from dammarenediol-II, and CYP716A53v2, which converts PPD to PPT—and UDP-glycosyltransferase (UGT)-catalyzed glycosylation [3,7,8,9,10,11,12]. Importantly, the steady-state accumulation of saponins *in planta* is determined not only by biosynthesis but also by degradation, transport and compartmentalized storage [3], and elicitation by chitosan or exogenous jasmonate can markedly enhance saponin accumulation through coordinated physiological and transcriptional responses [13,14]. In *Panax* leaves, a two-component chemical defense system in which β-glucosidases hydrolyze ginsenosides has recently been characterized, demonstrating that ginsenoside catabolism is enzymatically active in leaf tissue [15,16].

Leaf senescence is a highly regulated developmental process involving systematic reprogramming of gene expression, ordered degradation of cellular structures and remobilization of nutrients to sink organs [17,18,19]. The cysteine protease *SAG12* is a widely used molecular marker of senescence [20,21], and the bifunctional nuclease *BFN1* participates in nucleic acid degradation during senescence [22]. The execution of senescence-associated cell death engages vacuolar processing enzymes (VPEs), which mediate vacuolar collapse [23], metacaspases [24,25] and autophagy [25,26]; plant programmed cell death (PCD) during senescence is generally classified as a developmentally controlled, vacuolar-type process [27]. Senescence is orchestrated by layered hormonal and transcriptional networks: ethylene accelerates senescence through the EIN3-miR164-ORE1 regulatory module [28,29,30], salicylic acid (SA) promotes senescence via NPR1-dependent signaling [31,32], jasmonate (JA) signaling through MYC2 modulates the SA-JA balance that gates senescence onset [33,34], and the MKK9-MPK6 MAP kinase cascade transduces senescence signals [35]. At the transcription factor level, NAC proteins such as NAP/ORE1 and WRKY proteins such as *WRKY53* and *WRKY75* act as central positive regulators [36,37,38,39].

How senescence reshapes secondary metabolism has been systematically documented in *Arabidopsis*, where developmental senescence triggers coordinated spatiotemporal shifts across primary, secondary and lipid metabolism [40]. For medicinal plants, however, the coupling between leaf senescence and the accumulation of specialized metabolites remains largely unexplored [41]. In ginseng, leaves contain substantial ginsenosides and represent an important by-product of cultivation [42,43]; and although integrated transcriptomic-metabolomic analyses have proven powerful for resolving specialized-metabolite regulation in medicinal plants [44], no study has tracked ginsenoside dynamics across the senescence of ginseng leaves. Our preliminary profiling revealed a striking class-specific behavior: whereas PPT- and OT-type saponins increased monotonically during senescence, PPD-type saponins peaked at late senescence and then fell sharply at the wilted stage, a peak-and-decline pattern that raises two key questions. Does the senescence program itself drive the decline of PPD-type saponins at the final, frost-terminated stage, and how do senescence signals differentially regulate the accumulation of the different saponin classes?

To address these questions, we integrated four layers of analysis. First, we performed targeted metabolomic profiling of 376 saponin metabolite features across four sampling stages spanning the natural senescence progression (T1–T3) and a frost-terminated stage (T4), with temporal effects quantified by generalized estimating equations (GEE). Second, paraffin-section histology was used to validate senescence-associated cellular changes. Third, transcriptomic profiling of stages T1–T3 covered senescence core executors (metacaspases, VPEs, autophagy genes, *SAG12*), upstream signaling pathways (hormones, mitogen-activated protein kinase (MAPK), reactive oxygen species (ROS), sphingolipids) and transcription factor families (WRKY, NAC, auxin response factor (ARF) and basic helix-loop-helix (bHLH)). Finally, metabolome-transcriptome joint analysis linked saponin accumulation to senescence regulatory programs. We thereby establish a temporal atlas of ginsenoside accumulation during leaf senescence and propose a senescence–saponin regulatory network as a hypothesis-generating framework.

## 2. Results

### 2.1. Saponin Component Inventory, Detection Stability and Senescence-Driven Sample Separation

**Figure 2:**
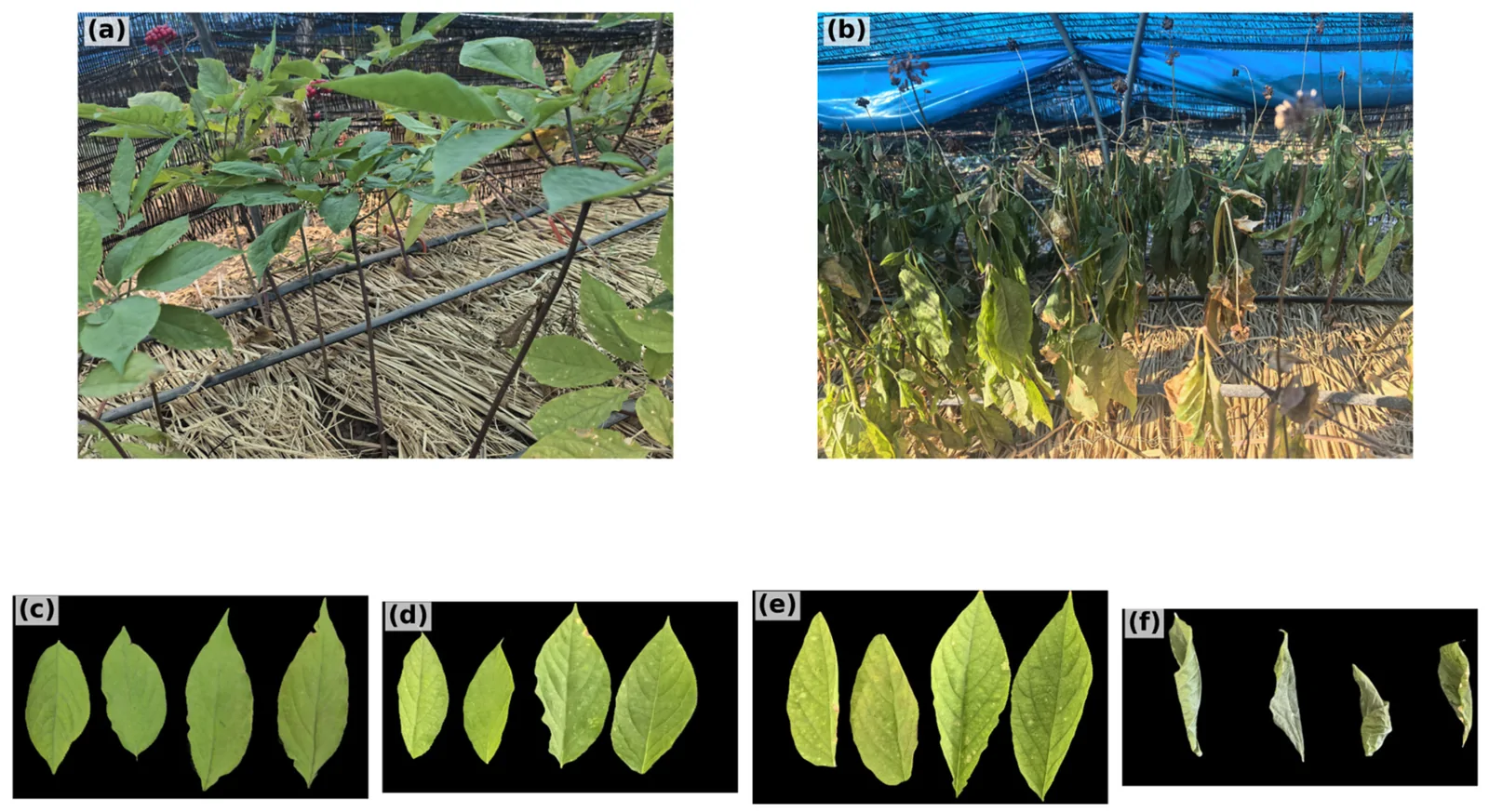
Field-grown plants and representative leaves at the four sampling stages. (**a**) Healthy three-year-old field-grown ginseng plants before senescence; (**b**) the same field after the acute cold snap (an approximately 10 °C temperature drop) that wilted and desiccated the canopy; (**c**–**e**) representative leaves at stages T1 (post-red-fruit, fully green), T2 (early senescence, yellowing initiating at leaf tips) and T3 (late senescence, approximately 50% of leaf area yellowed); (**f**) a frost-injured, wilted T4 leaf collected after the cold snap. T1–T4 denote the four sampling stages defined in Section 4.1.

**Figure 3:**
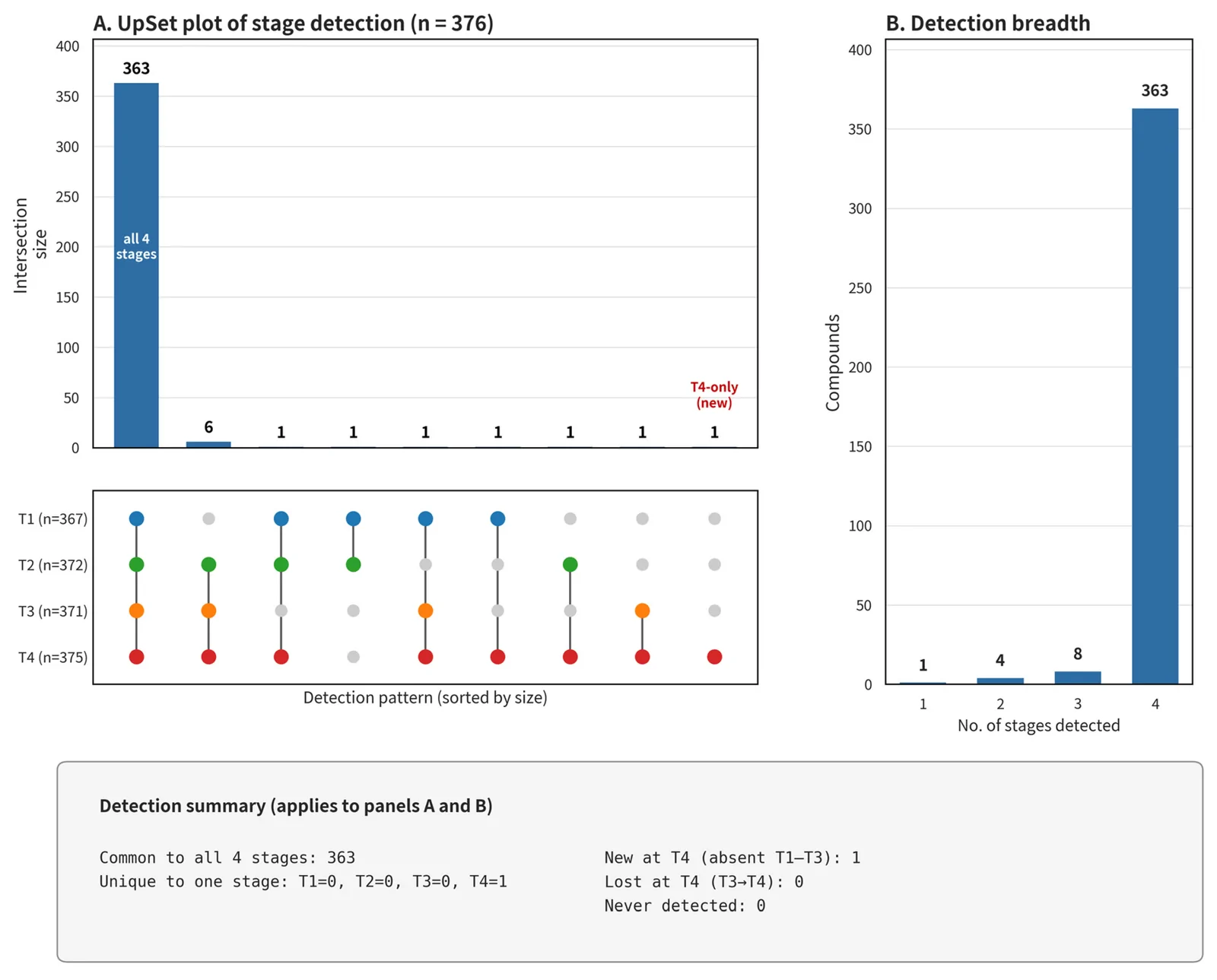
Detection analysis of the 376 saponin features across the four sampling stages. A compound was considered detected at a stage when at least two of three replicates showed non-missing abundance. (**A**) UpSet plot of stage-detection sets (T1–T4): 363 of 376 saponins (96.5%) were detected at all four stages; one compound appeared only at T4, and no compound detected at T3 was lost at T4. In the dot matrix, colored dots indicate detection at the corresponding stage (T1 blue, T2 green, T3 orange, T4 red) and gray dots indicate no detection. (**B**) Detection breadth: number of saponins detected at exactly one, two, three or all four stages. The summary box beneath the panels reports figure-level detection counts applying to both panels. Senescence therefore remodels the saponin metabolome quantitatively rather than qualitatively.

Targeted profiling detected 385 metabolite features in senescing ginseng leaves—376 saponin features (139 PPT-, 87 PPD-, 32 OT-, 40 OA-type and 78 variant-aglycone Other saponins) plus nine non-saponin small molecules (Table S2). Representative field plants and leaves at each of the four sampling stages are shown in Figure 2. The nine non-saponin small molecules were recorded in the detection inventory (Table S2) only and were not subjected to any temporal or accumulation-pattern analysis. The inventory spans one to five sugar moieties (median two to three per class; glucose the predominant sugar, followed by apiose/pentose and rhamnose) and comprises 259 unmodified glycosides together with 81 acetylated and 47 malonylated derivatives (categories not mutually exclusive), consistent in structural scope with the saponin diversity cataloged across the genus *Panax* and previously profiled in *Panax* leaves by LC/MS-based surveys [4,5,6,45]. Detection analysis showed that 363 of the 376 saponin features (96.5%) were consistently detected at all four stages, with no compound detected at T3 lost at T4 and only one newly appearing (Figure 3), indicating that senescence remodels the saponin metabolome quantitatively rather than through qualitative appearance or disappearance of components. The 13 features with incomplete detection comprised eight late-emerging compounds (six detected from T2, one from T3, and a single OA-2glc glycoside only at T4) and five intermittently detected (early-lost) compounds, mainly OA-type glycosides and variant-aglycone saponins whose abundance rose by one to two orders of magnitude over senescence; these compounds thus crossed the instrumental detection limit as they accumulated, rather than appearing through *de novo* synthesis (Table S3).

Two aspects of this classification deserve clarification. First, saponin identity is unambiguous for all 376 saponin features, including the variant-aglycone members: every feature was annotated through the diagnostic-fragmentation strategy established for ginsenoside profiling [46,47], exhibiting the defining signature of a triterpenoid glycoside—a deprotonated or formate-adducted high-mass precursor undergoing stepwise neutral losses of glycosyl residues (hexose 162 Da, pentose 132 Da, deoxyhexose 146 Da, together with acetyl 42 Da or malonyl 86 Da losses where applicable) down to a characteristic aglycone product ion. This fragmentation cascade is exclusive to saponins and establishes class identity even when the exact aglycone connectivity remains tentative. Second, the variant-aglycone group is intentionally broad. Its members deviate from the classical scaffolds through ring contraction or expansion, C17 side-chain rearrangement or backbone dehydrogenation; they occur at low individual abundance, are frequently isobaric—multiple positional or stereoisomers sharing identical *m*/*z* and near-identical fragment spectra—and lack authentic reference standards. Within the metabolomics identification-confidence framework [48], such features support confident class-level assignment but not reliable subdivision into structural subtypes, and a pooled group of this heterogeneity does not constitute a unified chemical entity amenable to class-level kinetic or correlation analysis. We therefore report the variant-aglycone saponins at the inventory level as supporting evidence and focus the mechanistic and temporal analyses on the four classical aglycone classes.

**Figure 4:**
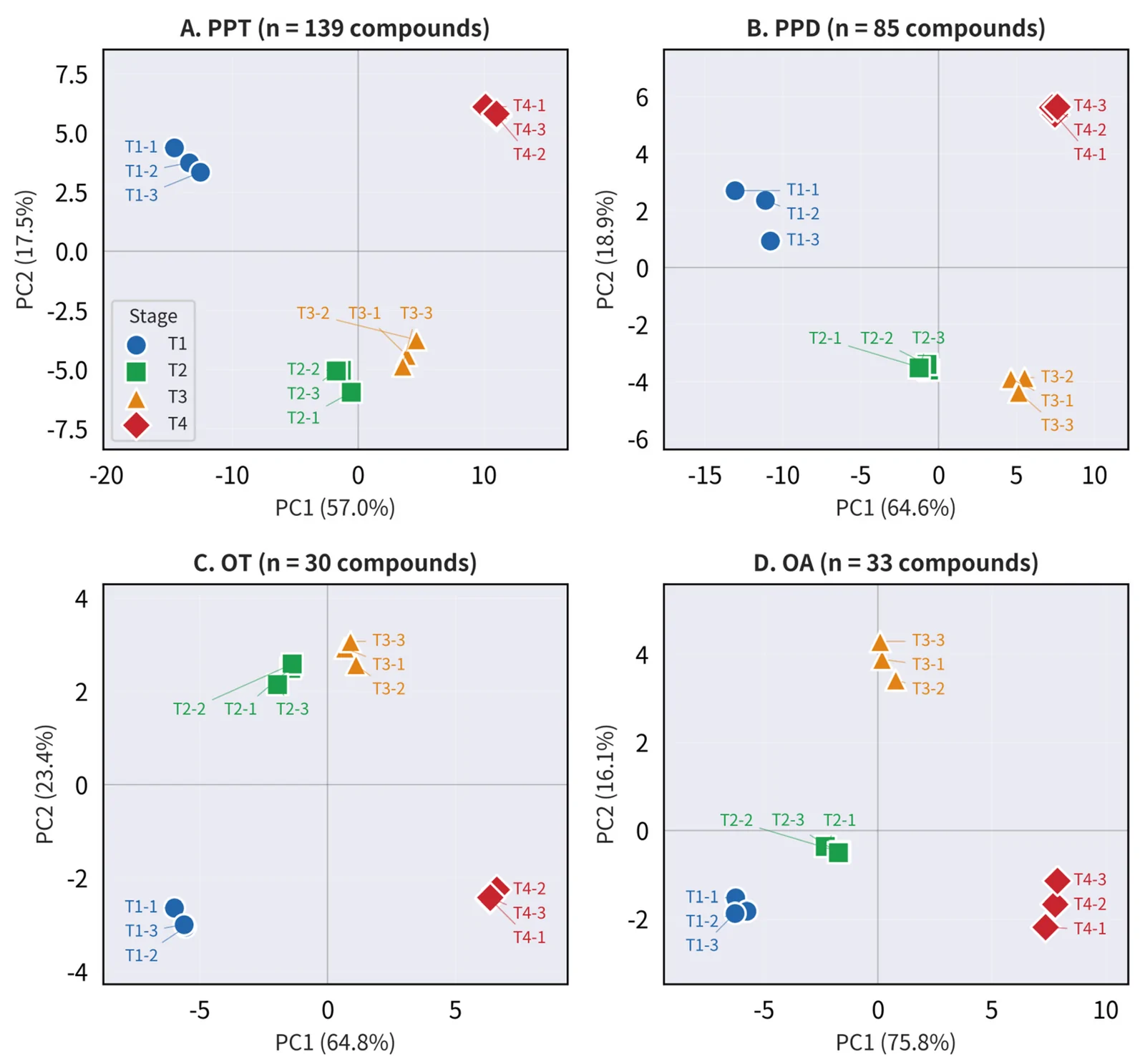
Principal component analysis of leaf saponin profiles per aglycone class. The analysis covered the 12 leaf samples (four stages × three replicates) and was computed on log_2_-transformed, z-scored compound abundances for the protopanaxatriol (**A**), protopanaxadiol (**B**), ocotillol (**C**) and oleanolic acid (**D**) classes. Samples separate along the first principal component in strict T1-to-T4 order with tight replicate clustering (variance explained by the first and second principal components: protopanaxatriol 57.0%/17.5% (n = 139), protopanaxadiol 64.6%/18.9% (n = 85, two zero-variance features removed), ocotillol 64.8%/23.4%, oleanolic acid 75.8%/16.1%), confirming senescence stage as the dominant driver of metabolic variation; the terminal displacement of T4 samples in the protopanaxadiol panel reflects the frost-associated decline of protopanaxadiol-type saponins.

PCA performed separately for each class confirmed that senescence stage is the dominant driver of metabolic variation: in all four aglycone-defined classes, samples separated along PC1 in strict T1-to-T4 order with tight replicate clustering (PC1 explaining 57.0–75.8% and PC2 16.1–23.4% of the variance across classes; Figure 4), the PPD panel showing a pronounced terminal displacement of T4 samples consistent with the frost-associated PPD decline described below.

### 2.2. Divergent Temporal Accumulation Programs of the Four Classical Saponin Classes

**Figure 5:**
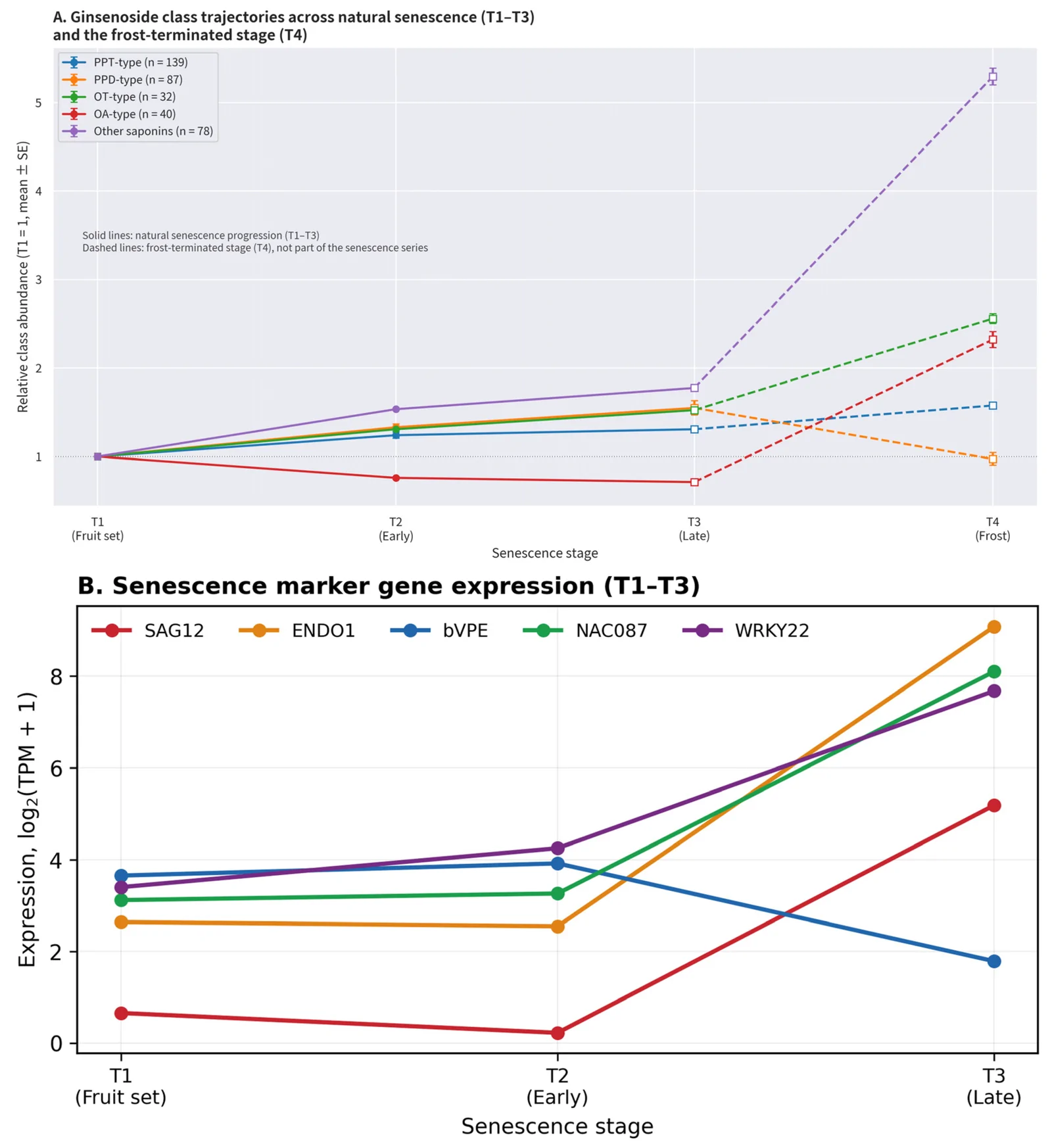
Temporal dynamics of saponin accumulation and senescence marker expression during ginseng leaf senescence. (**A**) Class-level, abundance-weighted trajectories of the five saponin groups (protopanaxatriol-type, n = 139; protopanaxadiol-type, n = 87; ocotillol-type, n = 32; oleanolic acid-type, n = 40; variant-aglycone Other saponins, n = 78; feature counts refer to the curated 376-saponin inventory), normalized to T1 = 1; error bars show the standard error across the three biological replicates. Solid lines trace the natural senescence progression (T1–T3); dashed lines connect T3 to the frost-terminated stage T4, which is not part of the senescence series. Protopanaxadiol-type saponins accumulate through T3 (abundance-weighted T3/T1 = 1.55) and fall back to the T1 level at the frost endpoint (abundance-weighted T4/T1 = 0.97); oleanolic acid-type saponins follow a U-shaped curve across T1–T3 and surge at T4; protopanaxatriol- and ocotillol-type saponins increase throughout, whereas Other saponins rise across T1–T3 and surge at T4. (**B**) Expression of senescence marker genes (log_2_(transcripts per million + 1), stages T1–T3): *SAG12*, *ENDO1*, *NAC087* and *WRKY22* are induced at T3, whereas the vacuolar processing enzyme gene *bVPE* is down-regulated (Welch’s *t*-test; T3 inferences are based on two qualified replicates and are hypothesis-level).

The five saponin groups exhibited markedly divergent accumulation trajectories across the natural senescence progression (T1–T3) and the frost-terminated stage (T4) (Figure 5A; Table 1 and Table 2). To separate natural senescence from frost effects, temporal trends were analyzed in two steps. First, GEE modeling restricted to the natural senescence gradient (T1–T3) converged for 374 of the 376 saponin features, and 326 of them (86.7%) showed significant temporal effects (FDR < 0.05; 224 increasing and 102 decreasing), demonstrating that natural senescence exerts a global influence on the saponin metabolome (feature-level GEE statistics are given in Table S4). All statistics reported in this section were computed on the curated set of 376 saponin features: the initial quantification matrix contained 410 features, from which nine non-saponin small molecules, 22 redundant non-saponin chromatographic peaks and three internal-standard peaks were excluded before analysis (Section 4.2), so that the analysis matrix is identical to the saponin inventory of Section 2.1. Significance rates were uniformly high across classes: protopanaxadiol-type 88.5% (77/87), protopanaxatriol-type 82.0% (114/139), ocotillol-type 90.6% (29/32), oleanolic acid-type 92.5% (37/40) and Other saponins 88.5% (69/78) (Table 1). Second, the frost-terminated stage T4 was compared with T3 as a distinct endpoint (feature-wise log_2_ fold change, Welch’s *t*-test with class-internal Benjamini–Hochberg FDR control). The frost endpoint remodeled the metabolome in a class-specific manner: median per-compound T4/T3 ratios were 0.97 for protopanaxadiol-type (42 significant features, 26 decreasing and 16 increasing), 1.21 for protopanaxatriol-type (86 significant, 66 increasing and 20 decreasing), 1.93 for ocotillol-type (28 significant, 22 increasing and 6 decreasing), 5.70 for oleanolic acid-type (33 significant, 26 increasing and 7 decreasing) and 2.27 for Other saponins (66 significant, 59 increasing and 7 decreasing).

We performed K-means clustering on the standardized temporal profiles (k = 2). Cluster assignments were derived from a clustering run on the cleaned data matrix; a total of 366 saponin profiles with complete standardized series were retained and assigned, and statistics were summarized over those 366 features. The saponins separated into two groups: a slow-rising group (Cluster 0, n = 231; 86.6% significant) and a steep-rising group (Cluster 1, n = 135; 89.6% significant). The mean temporal coefficient of Cluster 1 was 100.9-fold that of Cluster 0. The median ratio was 58.9-fold, confirming that the difference is not driven by extreme values (Table 2). At the class level, PPT- and OT-type saponins accumulated monotonically across the natural senescence progression (T1–T3) and continued to rise at the frost-terminated endpoint. Their abundance-weighted class-level T4/T1 ratios reached 1.58 and 2.56, respectively (Figure 5A; Figures S1 and S2). The variant-aglycone Other-saponin group likewise accumulated overall. It rose across the natural senescence progression (T1–T3; abundance-weighted class-level T3/T1 = 1.78) and surged at the frost-terminated endpoint (median of per-compound ratios T4/T3 = 2.27; abundance-weighted class-level T4/T1 = 5.29; Figure S4). OA-type saponins followed a U-shaped curve. Their abundance declined by 29% from T1 to T3 (abundance-weighted ratio 0.71) and then surged at the frost-terminated endpoint to 2.32 (abundance-weighted T4/T3 = 3.26; Figure S3). In striking contrast, PPD-type saponins accumulated continuously across natural senescence, peaked at T3 (abundance-weighted class-level T3/T1 = 1.55) and then declined by 37% at the frost-terminated endpoint (abundance-weighted class-level T4/T3 = 0.63), returning almost exactly to the T1 level (T4/T1 = 0.97). The PPD decline is therefore a frost-endpoint event rather than a feature of the natural senescence program. The two PPD endpoint statistics are consistent: the per-compound median T4/T3 is 0.97 (Section 2.2), whereas the abundance-weighted class-level T4/T3 is 0.63—the median compound is nearly unchanged, and the 37% loss of the class total shows that the decline is concentrated in high-abundance, low-glycosylation compounds (Section 2.3).

### 2.3. The Frost-Associated PPD Decline Is Glycosylation-Dependent

**Figure 6:**
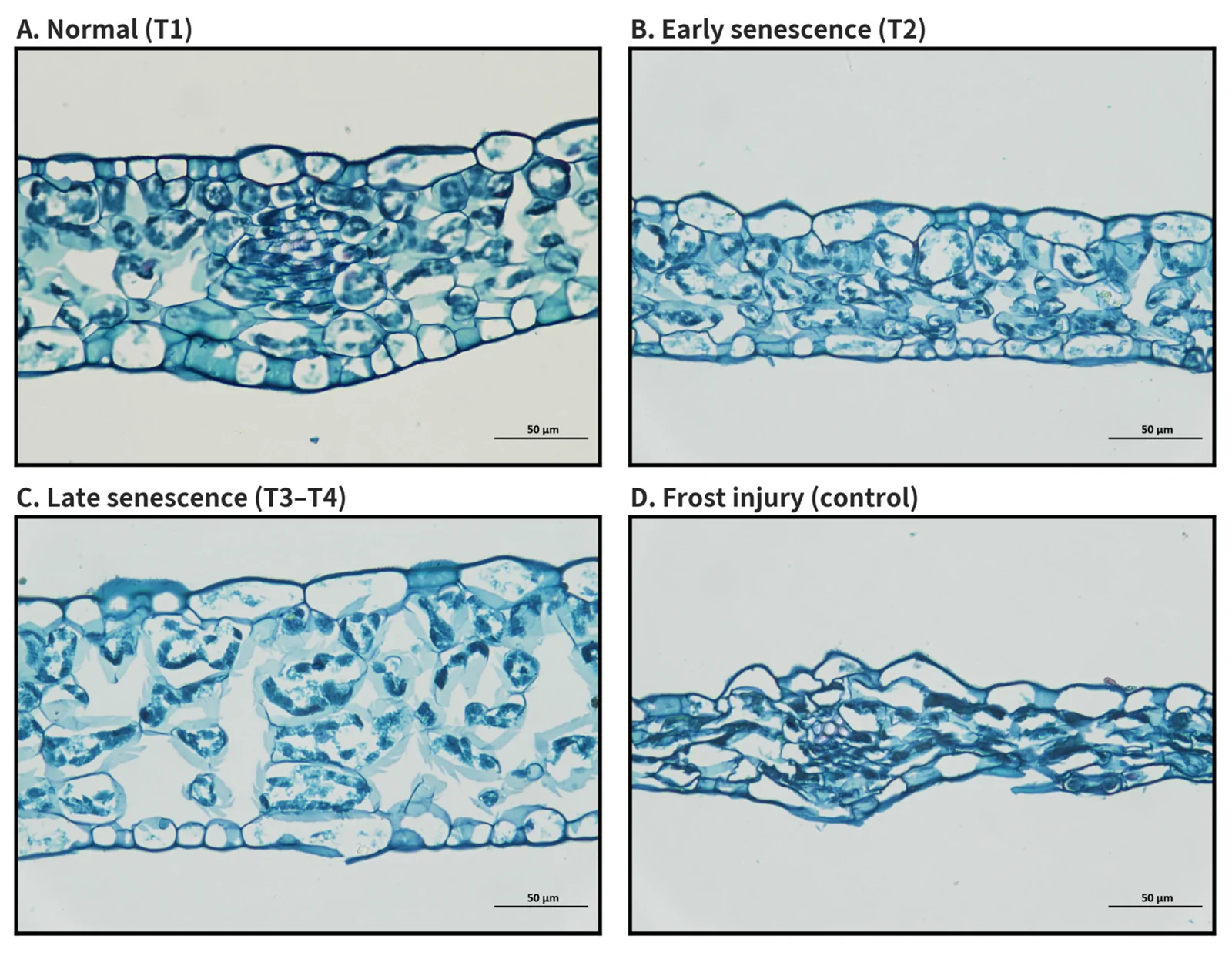
Histological validation of leaf senescence by paraffin-section microscopy (400×). (**A**) Healthy T1 leaf: compact epidermis, columnar palisade tissue rich in chloroplasts, regular spongy mesophyll. (**B**) Early senescence (T2): cell swelling and loosening, enlarged intercellular spaces, partial chloroplast disintegration. (**C**) Late senescence (T3–T4): loss of cell shape, extensive chloroplast breakdown, indistinct vacuolar boundaries indicating tonoplast collapse. (**D**) Frost-injured leaf (necrotic contrast): cell shrinkage, wall invagination and condensed contents, distinct from the ordered degradation of natural senescence. Panels (**A**,**D**) show sections through the vascular-bundle (midrib) region, whereas panels (**B**,**C**) show intercostal mesophyll regions; this difference in section position reflects the tissue integrity of the field-collected leaves at each sampling, and the diagnostic features of senescence (epidermal integrity, palisade organization, chloroplast abundance and vacuolar boundaries) are identifiable in both regions. Region-matched sections at every stage were not obtainable from the field-collected material, so regional effects cannot be fully excluded; the comparisons above are therefore made at the tissue level only.

**Figure 7:**
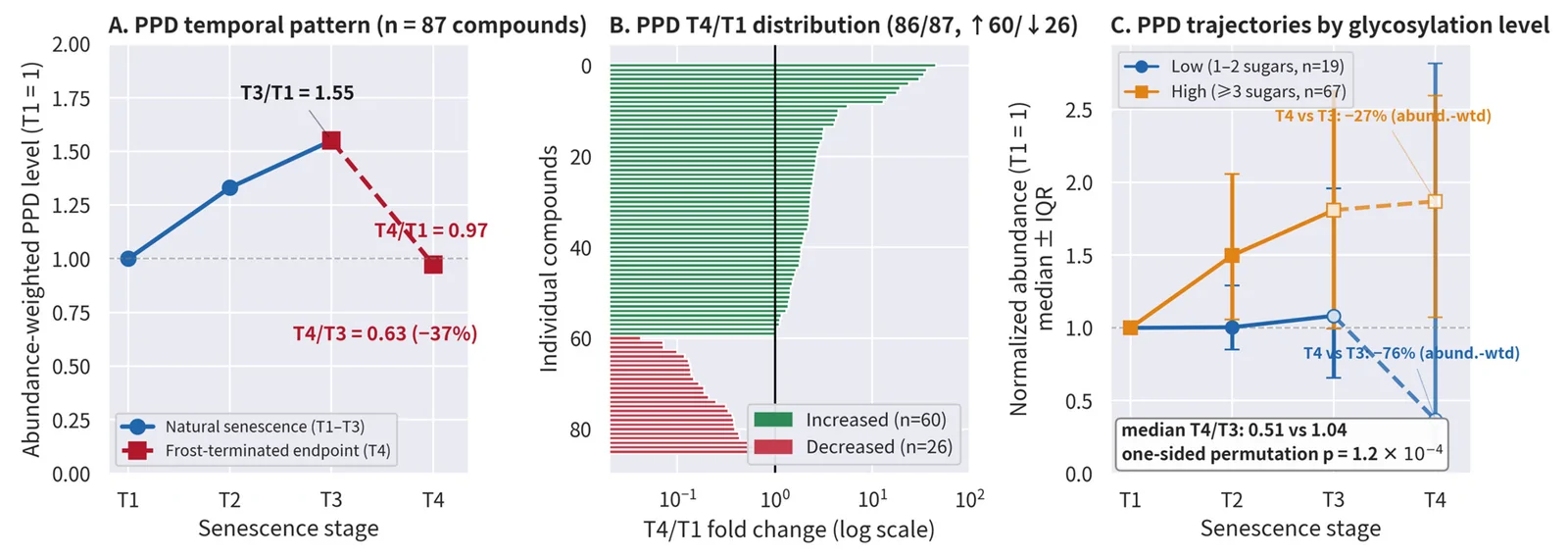
The frost-associated decline of protopanaxadiol (PPD)-type saponins is glycosylation-dependent. (**A**) Class-level, abundance-weighted trajectory: PPD-type saponins accumulate across natural senescence (T1–T3; T3/T1 = 1.55) and decline by 37% at the frost-terminated endpoint (T4/T3 = 0.63), returning to the T1 level (abundance-weighted T4/T1 = 0.97). (**B**) T4/T1 fold-change distribution of individual PPD-type compounds (86 of 87 features with complete four-stage series; green: 60 increased; red: 26 decreased). (**C**) Trajectories stratified by glycosylation degree: low-glycosylation PPD-type saponins (1–2 sugar moieties, n = 19) decline from T3 to T4 (median T4/T3 = 0.51), whereas highly glycosylated PPD-type saponins (≥3 sugars, n = 67) remain near their T3 level (median T4/T3 = 1.04; one-sided permutation test, *p* = 1.2 × 10^−4^ with 100,000 permutations).

Because the PPD decline occurred at the frost-terminated endpoint (histological evidence in Section 2.4 and Figure 6), we examined its compound-level structure (Figure 7). At the individual-compound level, 60 of the 86 PPD compounds with complete T1–T4 quantification (one of the 87 PPD-type features lacked a full four-stage series) still ended higher at T4 than at T1, yet the abundance-weighted class total was dominated by the strong T3-to-T4 drop (Figure 7A,B). Stratification by glycosylation degree resolved this pattern: low-glycosylation PPD compounds (one to two sugar moieties, n = 19) fell to a per-compound median T4/T3 of 0.51 (abundance-weighted T4/T3 = 0.24), whereas highly glycosylated compounds (three or more sugars, n = 67) remained near their T3 level (per-compound median T4/T3 ratio 1.04; abundance-weighted T4/T3 = 0.73; one-sided permutation test on the difference in medians, *p* = 1.2 × 10^−4^ with 100,000 permutations; a sensitivity split at three or fewer versus four or more sugars was not significant, *p* = 0.15; Figure 7C). This glycosylation-dependent decline is consistent with deglycosylation-mediated catabolism: β-glucosidases, which are active in *Panax* leaves [15,16], can strip sugar moieties from PPD glycosides, and the resulting aglycones are far less water-soluble and more prone to oxidative degradation. Our transcriptome data are consistent with this mechanism, although the two-replicate T3 transcriptome makes this support hypothesis-level: multiple β-glucosidase genes were strongly induced at T3 (e.g., BGLU12/EVM0054313, TPM 55.5 to 299.3; EVM0052430, 122.1 to 360.3; EVM0024249, 4.3 to 43.1), consistent with senescence-associated activation of deglycosylation capacity. Family-wide, 21 of the 123 annotated β-glucosidase genes were induced at T3 (|log_2_FC| > 1; mean log_2_FC = +3.9) and 51 were repressed (mean log_2_FC = −3.5), 14 and 40 of them significant at FDR < 0.05, respectively; however, the induced set comprises the predominantly expressed members—its aggregate TPM rose 3.9-fold (192 to 741) while the repressed, low-abundance set fell from 317 to 60 TPM—so total family expression increased by 45% at T3, pointing to a net gain of deglycosylation capacity at the execution phase (Table S6). The UDP-glycosyltransferase (UGT) family showed the complementary response: 103 of 242 annotated glycosyltransferase genes were repressed at T3 (91 at FDR < 0.05; mean log_2_FC = −3.3), against 29 induced (26 at FDR < 0.05; mean log_2_FC = +2.8), and aggregate expression of the repressed set fell 3.9-fold (1510 to 390 TPM). Concurrent repression of glycosylation capacity and induction of deglycosylation capacity at the execution phase thus offers a candidate-level transcriptional explanation, requiring validation with larger replicate numbers, for the preferential loss of low-glycosylation PPD compounds. Notably, no free ginsenoside aglycones were detected at any stage, consistent with rapid further conversion of deglycosylated products. Loss of vacuolar compartmentalization during senescence execution (Section 2.4) would bring stored PPD saponins into contact with such hydrolases. We emphasize that this deglycosylation–vacuolar-exposure mechanism is a hypothesis supported by convergent but correlative evidence (temporal coupling, glycosylation-dependent stratification and transcript-level β-glucosidase induction); validation at the enzyme-activity, protein and subcellular-localization levels is still required (Section 3.6).

### 2.4. Histological Validation of Ordered Senescent Degradation

Paraffin-section microscopy at 400× confirmed the histological progression of senescence (Figure 6). Healthy T1 leaves showed tightly arranged upper and lower epidermis, columnar palisade cells densely packed with chloroplasts, and spongy mesophyll with regular intercellular spaces (Figure 6A). Early-senescent leaves (T2) displayed cell swelling and loosening, enlarged intercellular spaces, thinned cytoplasm and partial chloroplast disintegration (Figure 6B). Late-senescent leaves (T3–T4) showed extensive structural breakdown: cells lost their regular shape, chloroplasts were largely disassembled, and vacuolar boundaries became indistinct, indicating loss of vacuolar membrane integrity (Figure 6C). In contrast, frost-injured leaves exhibited cell shrinkage, wall invagination and condensed, intensely stained contents—an acute necrotic-damage pattern clearly distinct from the ordered, swelling-type degradation of natural senescence (Figure 6D). These observations corroborate that the senescing ginseng leaf undergoes regulated, PCD-like degradation rather than necrotic death [27], and they temporally align the vacuolar collapse proposed to expose PPD saponins to catabolic enzymes with the T3–T4 window in which the PPD decline occurs. Regarding comparability across panels, the field-collected senescing leaves were sectioned where tissue integrity was best preserved: panels A and D show the vascular-bundle (midrib) region, whereas panels B and C show intercostal mesophyll; epidermis, palisade tissue and spongy mesophyll are identifiable in both regions, so the stage-specific features described above are comparable at the tissue level. Nevertheless, because the sectioned region could not be standardized across stages in the field-collected material, region-level differences between midrib and intercostal tissue cannot be fully excluded as a contributor to the stage contrasts; the stage-specific interpretations above are therefore restricted to features identifiable in both regions, and region-matched serial sectioning will be required to fully exclude regional effects.

### 2.5. Global Transcriptomic Reprogramming at Late Senescence

Comparison of the T3 (late senescence) and T1 (pre-senescence) transcriptomes revealed large-scale reprogramming: of 56,128 testable transcripts, 26,632 (47.4%) were down-regulated and 4740 (8.4%) up-regulated at the dual threshold (|log_2_FC| > 1 and FDR < 0.05), a 5.6-fold asymmetry toward repression. This pattern is characteristic of the senescence execution phase, in which ribosome biogenesis, photosynthetic and chloroplast-related genes are shut down en masse while a smaller set of degradation, autophagy and nutrient-remobilization genes is selectively induced [17,18,40,49]. The transcriptomic timing coincides with the histological features (chloroplast disintegration, vacuolar collapse) observed at T3 (Figure 6C). These differential-expression statistics are based on six qualified T1 replicates versus two qualified T3 replicates and are therefore interpreted as an indicative overview of senescence-associated reprogramming rather than as definitive quantification.

### 2.6. Sequential Activation of Senescence Core Executors

Senescence core executor genes showed differentiated temporal activation (Figure 5B; Table 3). Among 18 annotated metacaspase genes, expression was isoform-specific: one AMC1 copy was strongly induced at T3 (log_2_FC = +1.61; TPM 12.4 to 37.7) whereas another was repressed (log_2_FC = −2.62), and an AMC4 copy was up-regulated (log_2_FC = +1.11), suggesting that type-II metacaspases contribute differentially to senescence execution [24,25]. The vacuolar processing enzyme *bVPE* was significantly down-regulated at T3 (log_2_FC = −2.24; TPM 11.6 to 2.5); because VPE-mediated vacuolar processing precedes tonoplast collapse [23], this late-stage repression is compatible with feedback shutdown after vacuolar breakdown has been initiated. In contrast, the senescence-specific cysteine protease *SAG12* was sharply induced (log_2_FC = +5.94; TPM 0.6 to 35.4), and the nuclease *ENDO1*, a functional counterpart of *Arabidopsis BFN1* [22], rose from 5.2 to 538.8 TPM (log_2_FC = +6.69). The coordinated induction of *SAG12* and *ENDO1* is consistent with T3 marking the onset of the irreversible degradation phase—the same window in which PPD-type saponins begin the decline that is completed at the frost-terminated endpoint. Autophagy genes were broadly repressed: one ATG5 copy fell to undetectable levels (log_2_FC = −9.85), and single copies of ATG10 and ATG16 were likewise silenced (log_2_FC = −9.06 and −7.26), consistent with a shift from selective autophagic maintenance to wholesale cellular degradation [26]. Because the T3 transcriptome comprises two qualified replicates, these executor-expression patterns are interpreted as consistent with senescence execution at T3 rather than as definitive evidence; confirmation with larger replicate numbers is required.

### 2.7. Differential Responses of Upstream Senescence Signaling Pathways

**Figure 8:**
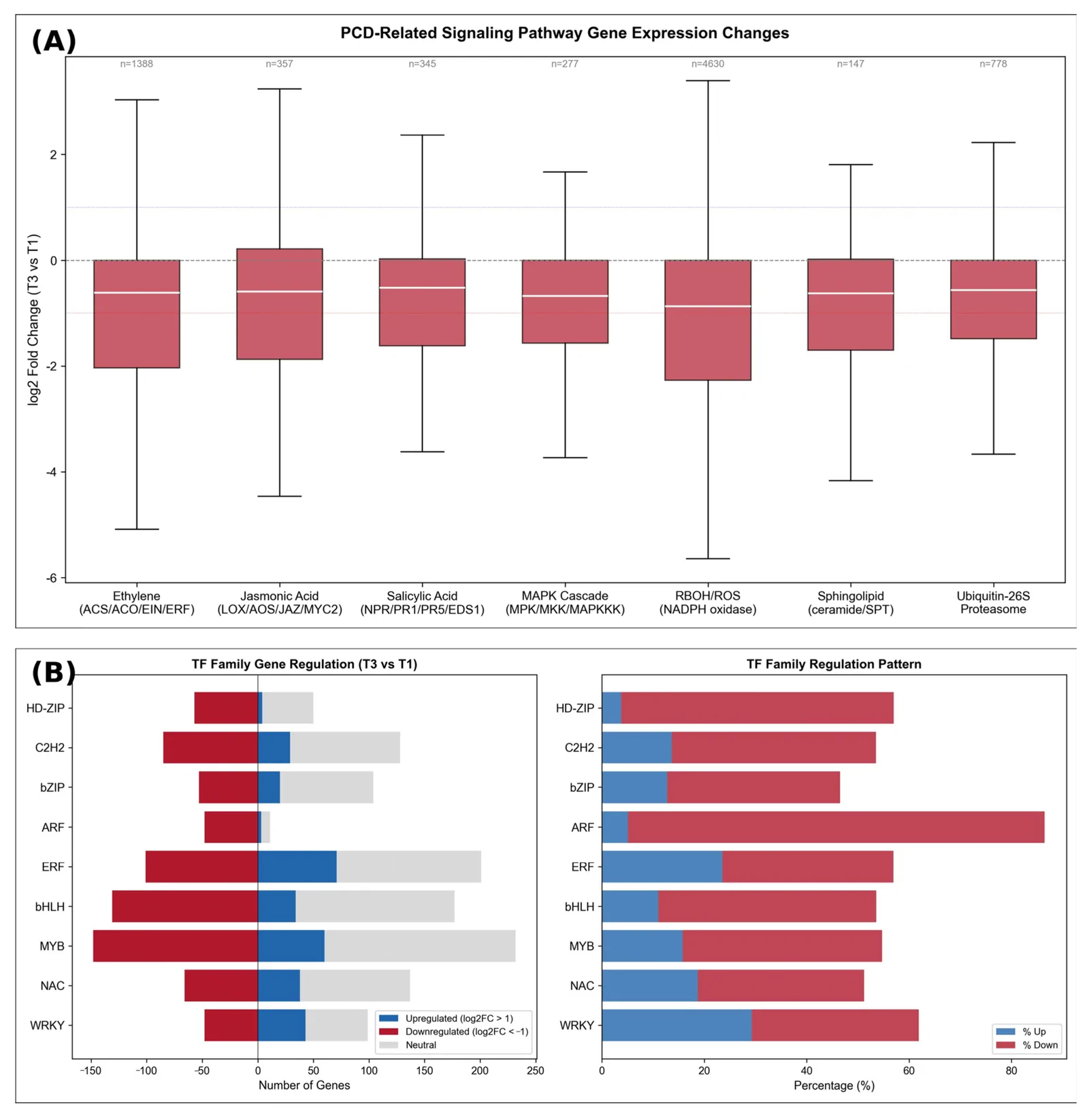
Upstream senescence signaling and transcription factor family responses (T3 vs. T1). (**A**) Expression changes in senescence-related signaling pathway genes: distributions of log_2_ fold changes per pathway; dashed lines mark the 2-fold up- (blue) and down-regulation (red) thresholds. Salicylic acid signaling is activated (the pathogenesis-related marker PR1B1 is strongly induced), jasmonate signaling is strongly repressed (the transcription factor MYC2 is silenced), and ethylene and mitogen-activated protein kinase core components are predominantly down-regulated. (**B**) Transcription factor families show opposed regulatory biases: numbers of up- and down-regulated genes per family (direction of the log_2_ fold change, left) and corresponding percentages (right). WRKY (43% up), ERF (34% up) and NAC (29% up) show upward biases, whereas ARF (88% down), HD-ZIP (72% down) and bHLH (58% down) are predominantly repressed. Expression changes at T3 are based on two qualified replicates and are presented as hypothesis-level patterns.

Upstream signaling pathways responded asymmetrically (Figure 8A; Table 3). Salicylic acid signaling was activated: pathogenesis-related markers were strongly induced at T3 (PR1B1 EVM0052323, TPM 1.8 to 129.4, FDR = 1.2 × 10^−6^; PR2 homologs predominantly up-regulated), echoing the senescence-promoting role of SA [32,34]. Jasmonate signaling was markedly repressed: two MYC2 copies dropped below detection (log_2_FC = −11.14 and −10.17; FDR < 1 × 10^−16^), and the JA-biosynthetic gene AOS1 was down-regulated (log_2_FC = −3.05; FDR = 1.5 × 10^−33^) while LOX genes responded heterogeneously, consistent with release of JA-mediated antagonism of senescence-promoting factors [33,34]. Ethylene-pathway genes were predominantly down-regulated (237 of 595 down, 39.8%, of which 208 were significant at FDR < 0.05; only 87 up, 14.6%, of which 68 were significant), suggesting attenuation of ethylene sensitivity during the execution phase, in line with feedback turnover of EIN3/EIL1 signaling [28,29]. Calcium signaling components were likewise broadly suppressed: of 213 annotated Ca^2+^-signaling genes (calmodulins, CPKs, CBLs and CIPKs), 114 changed by |log_2_FC| > 1 at T3, with 90 down-regulated versus 24 up-regulated (81 and 17 of them significant at FDR < 0.05, respectively), including silencing of several CPK and CAM copies, suggesting attenuation of Ca^2+^-relay capacity during senescence execution. This broad suppression is not necessarily paradoxical: Ca^2+^ signaling acts biphasically in leaf senescence—promoting age-dependent and ABA-linked senescence through CaM1/CaM4 and CPK-ORE1 modules but restraining MeJA- and NO-mediated senescence in other contexts [50,51]—so transcriptional dampening of Ca^2+^ relays after execution onset is consistent with feedback attenuation once the phosphorylation events initiating senescence-associated cell death have occurred [51]. Within the MAPK cascade, MPK3 and MPK10 fell to undetectable levels (log_2_FC = −10.21 and −11.13; FDR < 1 × 10^−6^) while MKK9 was up-regulated at T3 (TPM 44.0 to 114.9; FDR = 5.3 × 10^−23^), recalling the MKK9-MPK6 module implicated in *Arabidopsis* senescence [35] and suggesting a handover from MAPK relay to direct transcriptional control. Sphingolipid metabolism was remodeled: dihydroceramide fatty acid hydroxylases (FAH1/FAH2) were repressed (log_2_FC = −1.8 to −4.0; FDR < 1 × 10^−12^) while serine palmitoyltransferase subunits remained essentially unchanged, whereas the glucosylceramide synthase GINT1 was newly expressed (TPM 0 to 0.72; FDR = 2.0 × 10^−13^), pointing to remodeling of complex sphingolipid species during membrane breakdown [52]. With only two qualified T3 replicates, these signaling asymmetries are underpowered and are presented as hypothesis-level patterns to be tested with larger sample sizes, and all pathway-level inferences above are candidate-level.

### 2.8. Transcription Factor Families Show Opposed Regulatory Biases

Among nine major transcription factor families, WRKY, ERF and NAC showed upward expression biases, whereas ARF, HD-ZIP and bHLH were predominantly repressed (Figure 8B). The WRKY bias was most striking: of 147 WRKY genes, 63 (43%) increased in expression (log_2_FC > 0) against 73 (50%) that decreased, and several individual WRKYs were strongly induced at T3—*WRKY22* (log_2_FC = +4.41; TPM 9.6 to 203.1), WRKY9 (+7.54; 0.2 to 43.7), WRKY72A (+4.70) and WRKY6 (+4.19). WRKY factors are established positive regulators of leaf senescence, exemplified by *WRKY53* and *WRKY75* in *Arabidopsis* [34,36,38,39], and the ginseng WRKY induction pattern is consistent with a comparable role. Within the NAC family, a homolog of *Arabidopsis ANAC087*—a regulator of developmental PCD [53]—was strongly induced (log_2_FC = +5.15; TPM 7.7 to 272.7), as was a NAC073 copy (log_2_FC = +5.21), nominating *NAC087* as a candidate senescence regulator in ginseng. Conversely, MYC2 (bHLH) silencing mirrored JA-pathway repression, and the strong downward bias of the ARF family (52 of 59 genes decreased, 88%) suggested shutdown of auxin signaling, an anti-senescence pathway whose closure marks the irreversible phase of senescence [17,18]. These family-level biases rest on two qualified T3 replicates and are consistent with, but do not by themselves establish, the proposed regulatory roles. All family-level inferences are exploratory and require validation with larger replicate numbers.

### 2.9. Joint Analysis Reveals a Dichotomous Regulatory Pattern

**Figure 9:**
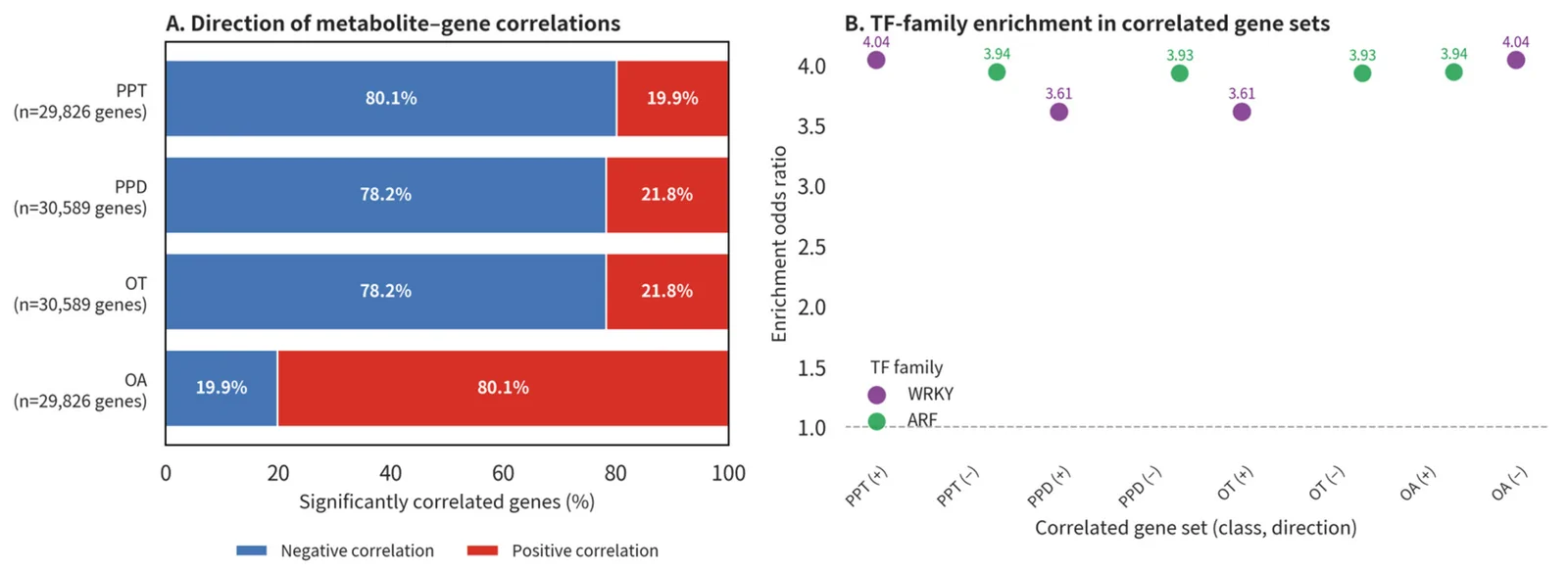
Metabolome–transcriptome joint analysis reveals a dichotomous regulatory pattern. (**A**) Direction of genes significantly associated with each of the four classical aglycone classes (protopanaxatriol (PPT), protopanaxadiol (PPD), ocotillol (OT) and oleanolic acid (OA); the structurally heterogeneous variant-aglycone Other-saponin group was excluded because its class-averaged profile does not represent a unified chemical entity); Spearman correlation on the 9-point stage-replicate matrix, |r| > 0.3, false discovery rate < 0.05; independent information derives from three stage pairs. Protopanaxatriol-, protopanaxadiol- and ocotillol-type accumulation is negatively correlated with ~80% of associated genes, whereas oleanolic acid-type accumulation is positively correlated with ~80%. (**B**) Transcription factor enrichment within class-associated gene sets (Fisher’s exact test): WRKY factors are enriched among PPT/PPD/OT-positive and OA-negative gene sets (odds ratio 3.61–4.04); ARFs show the mirror pattern (odds ratio 3.93–3.94). (n = 9 points: 3 stages × 3 metabolomic replicates; within-stage replicates are not independent time points, so the analysis is exploratory and inference rests on stage-level direction patterns; see Section 4.6).

Correlating class-level saponin abundance with gene expression across the nine replicate profiles (T1–T3) for the four classical aglycone classes (PPT, PPD, OT and OA) uncovered a clear dichotomy in this exploratory analysis (Figure 9A; the nine-point matrix derives from only three independent stages, so the associations are hypothesis-generating rather than confirmatory; Section 4.6); the variant-aglycone Other-saponin group was excluded from this analysis because its high structural heterogeneity means that the class-averaged profile does not represent a unified chemical entity. Among significantly associated genes, PPT-, PPD- and OT-type accumulation was negatively correlated with approximately 80% of associated genes (80.1%, 78.2% and 78.2%, respectively)—that is, these saponins accumulated while most co-varying genes were repressed—whereas OA-type accumulation was positively correlated with 80.1% of associated genes. This dichotomy suggests two distinct accumulation logics: passive accumulation of PPT/PPD/OT saponins as their turnover machinery is repressed, versus co-regulated production of OA saponins alongside senescence-activated genes.

Transcription factor enrichment analysis connected this dichotomy to the senescence regulatory layer (Figure 9B). WRKY factors were significantly enriched among genes positively correlated with PPT/PPD/OT classes and among genes negatively correlated with the OA class (OR 3.61–4.04; FDR < 0.05)—i.e., WRKY expression tracks the PPT-like accumulation program. ARFs showed the mirror image, enriching among genes negatively correlated with PPT/PPD/OT (OR 3.93–3.94). These enrichments point to WRKY as a candidate bridge between senescence signaling and saponin biosynthetic gene expression. This interpretation is consistent with the upward bias of WRKY expression at T3. Conversely, the repression of ARFs may relieve the auxin-mediated constraint on saponin accumulation.

### 2.10. A Working Model of Senescence–Saponin Regulatory Coupling

**Figure 10:**
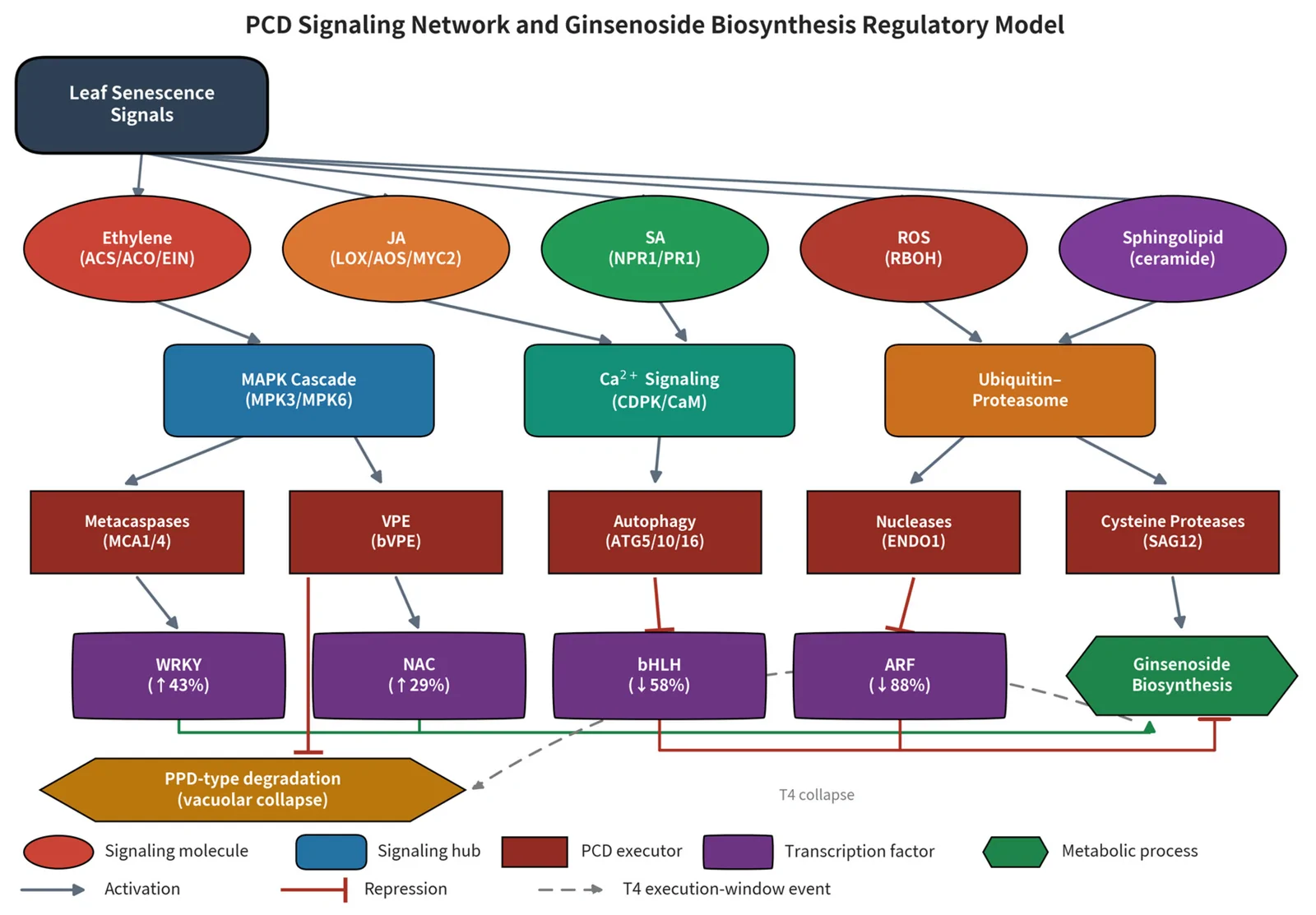
Working model (hypothesis) of the senescence signaling network regulating ginsenoside accumulation. Senescence signals are proposed to be relayed through five parallel signaling channels (ethylene, jasmonate, salicylic acid, reactive oxygen species and sphingolipid) to mitogen-activated protein kinase, Ca^2+^ (broadly down-regulated at T3) and ubiquitin–proteasome hubs, reshaping the transcription factor layer (WRKY and NAC induced; bHLH and ARF repressed). In this model, the transcription factor layer is proposed to drive (i) programmed cell death executors (metacaspases, vacuolar processing enzymes, autophagy, nucleases and cysteine proteases) and (ii) modulation of ginsenoside biosynthesis; during T3–T4, vacuolar collapse is hypothesized to expose compartmentalized protopanaxadiol-type saponins to glycosidase-mediated degradation, producing the PPD decline observed at the frost-terminated endpoint. This deglycosylation-exposure link is a hypothesis that awaits enzyme-activity and protein-level validation. Node shapes indicate node categories: ellipses, signaling molecules; rounded boxes, signaling hubs; square boxes, programmed cell death executors; beveled boxes, transcription factors; hexagons, metabolic processes. Arrows: activation; T-bars: repression. The dashed arrow marks the proposed event at the frost-terminated T4 stage.

Integrating the metabolomic, histological and transcriptomic evidence, we propose a working model of senescence–saponin coupling (Figure 10). Senescence signals are relayed through five parallel signaling channels (SA activation, JA repression and ethylene modulation, together with ROS and sphingolipid signaling) to MAPK and Ca^2+^ signaling hubs (the latter broadly down-regulated at T3), which reshape the transcription factor layer (WRKY and NAC induced; ARF and bHLH/MYC2 repressed). In this model, the transcription factor layer is proposed to drive two outputs: (i) activation of senescence executors (*SAG12*, *ENDO1*, metacaspases), producing the ordered cellular degradation observed histologically; and (ii) modulation of saponin biosynthetic flux. During the T3–T4 window, in which natural senescence execution is overtaken by frost termination, vacuolar collapse is hypothesized to expose compartmentalized PPD-type saponins to glycosidases, preferentially degrading low-glycosylation compounds and thereby contributing to the PPD decline observed at the frost-terminated endpoint, whereas PPT- and OT-type saponins—protected by glycosylation patterns, subcellular localization or continued biosynthesis—accumulate through T3 and, for most classes, into the frost-terminated stage.

## 3. Discussion

### 3.1. Coupling of the Frost-Endpoint PPD Decline to Senescence Execution

The central finding of this study is the temporal coupling between the continuous accumulation of PPD-type saponins across natural senescence (T1–T3) and the execution phase of leaf senescence, followed by a frost-associated PPD decline at the terminated endpoint. PPD accumulation rose continuously through T3 (T3/T1 = 1.55)—precisely when *SAG12* and *ENDO1* reached their highest expression among the profiled T1–T3 stages—and then fell by 37% at the frost-terminated endpoint to the T1 level (abundance-weighted class-level T4/T3 = 0.63; T4/T1 = 0.97). Because T4 transcriptomes could not be obtained, we cannot directly track gene expression during this window; nevertheless, the molecular onset of senescence execution at T3, together with the histologically verified loss of vacuolar integrity at T3–T4, provides a coherent, hypothesis-level mechanistic frame. Senescence-coupled reorganization of specialized metabolism is emerging as a general theme in root- and rhizome-crop species: in American ginseng (*Panax quinquefolius*), elevated temperatures accelerated leaf senescence while increasing root ginsenoside concentrations [54], and seasonal sampling of 2- to 4-year-old plants documented coupled declines of leaf weight and shifts in ginsenoside content as the canopy senesced [55], with ginsenoside content also varying with plant developmental stage in this congener [56]; in the medicinal herb *Artemisia annua*, artemisinin accumulates in leaf trichomes up to the onset of senescence and declines as senescence progresses [57]; and saponin accumulation in the roots of *Panax notoginseng* is likewise developmentally stage-dependent [58]. A shared pattern is therefore that leaf senescence in perennial root crops couples nutrient resorption [59] with a reallocation of specialized metabolism; the glycosylation-dependent, class-selective frost-endpoint decline of PPD-type saponins resolved here appears to be a distinctive feature of ginseng leaf senescence. Saponins are generally believed to be sequestered in vacuoles [3], although direct subcellular evidence in ginseng remains lacking; under this working hypothesis, tonoplast collapse during vacuolar-type PCD [23,27] would release stored PPD glycosides into the cytosol and expose them to β-glucosidases, which are enzymatically active against ginsenosides in *Panax* leaves [15,16] and whose genes are strongly induced at T3 in our transcriptome (Section 2.3). The preferential decline of low-glycosylation PPD compounds (per-compound median T4/T3 0.51 vs. 1.04 for highly glycosylated ones) is consistent with what this mechanism predicts: sparsely glycosylated molecules can be fully deglycosylated to unstable aglycones, whereas heavily glycosylated ones retain partial glycosylation and water solubility, and may additionally be exported via the phloem to sink organs such as roots. The frost-endpoint decline therefore probably blends catabolism with source-sink remobilization rather than reflecting degradation alone [60,61]. From the perspective of accumulation dynamics, the divergent class trajectories indicate that the peak window for leaf PPD-type saponin accumulation lies around T3, whereas PPT-, OT- and OA-type contents are highest in the frost-terminated T4 material. We note, however, that leaf biomass, total saponin yield per plant, harvesting cost and field feasibility were not measured in this study; any practical harvest-timing recommendation therefore requires agronomic trials that integrate these factors, and the present results speak only to metabolite-level accumulation windows.

### 3.2. Conservation of the Senescence Regulatory Module in Ginseng

The ginseng senescence transcriptome recapitulates much of the *Arabidopsis* senescence program: massive repression of housekeeping transcription, strong *SAG12* and nuclease induction, SA activation coupled to JA repression, and WRKY/NAC-centered transcriptional control [17,18,34]. Two observations deserve emphasis. First, the strong induction of a *NAC087* homolog (log_2_FC = +5.15) nominates it as a candidate senescence regulator in ginseng; in *Arabidopsis*, *ANAC087* controls developmental PCD in root-cap tissues [53], so a role in leaf senescence remains to be demonstrated and should be regarded as a hypothesis. Second, the WRKY family showed a pronounced upward expression bias (43% of 147 genes), with *WRKY22*, WRKY9, WRKY72A and WRKY6 strongly induced. Although *Arabidopsis WRKY22* is not itself a recognized senescence marker, the family’s senescence-promoting function is well established through *WRKY53* and *WRKY75* [36,39], and our data are consistent with functional conservation at family level. These candidates are hypothesis-level leads whose validation will require larger replicate numbers and functional assays.

### 3.3. A Dichotomous Regulatory Logic and a Dual Role for WRKY

The joint analysis revealed that roughly 80% of genes associated with PPT/PPD/OT accumulation were negatively correlated, whereas roughly 80% of genes associated with OA accumulation were positively correlated. We interpret the negative mode as passive accumulation: as global transcription—including turnover and catabolic machinery—shuts down, metabolically stable saponins persist and concentrate. The positive mode instead suggests active co-regulation with senescence-induced programs. WRKY enrichment within the PPT/PPD/OT-positive and OA-negative gene sets (OR 3.61–4.04) nominates WRKY factors as candidate mediators at the interface of the two modes: they may both drive senescence execution and modulate saponin pathway genes, a dual role analogous to WRKY integration of immune signaling and metabolic reprogramming in other species. Direct evidence—for example, W-box occupancy at CYP716A53v2 or UGT promoters—is required to substantiate this model. These correlations are exploratory: the nine-point matrix derives from only three independent senescence stages, and within-stage replicates are not independent observations, so the joint analysis is used to generate hypotheses rather than to confirm regulatory causality (Section 4.6).

### 3.4. Variant-Aglycone Saponins as Products of Aglycone-Level Scaffold Diversification

Beyond the four classical aglycone classes, the 78 variant-aglycone Other saponins carry putative aglycones that deviate from the classical PPT/PPD scaffolds by ring contraction or expansion, C17 side-chain rearrangement or backbone dehydrogenation, as assigned by diagnostic-fragment annotation (Section 4.2). Treating these variants as products of aglycone-level scaffold diversification is biosynthetically grounded: in triterpenoid saponin biosynthesis, the carbon skeleton is fixed at the aglycone-formation stage, when oxidosqualene cyclases cyclize 2,3-oxidosqualene into distinct scaffolds [62,63], before cytochrome P450-mediated oxidation [7,8] and UGT-catalyzed glycosylation [5] elaborate the mature saponins [3]; scaffold variants therefore arise as independent biosynthetic products rather than as derivatives of preformed ginsenosides. Because aglycone-scaffold isomerization of intact saponins has so far been documented mainly during post-harvest processing [64], and our leaves were flash-frozen in liquid nitrogen immediately after collection, a processing origin for the variant aglycones detected here can be excluded. As a group, these saponins rose across the natural senescence progression (T1–T3; abundance-weighted class-level T3/T1 = 1.78) and surged at the frost-terminated endpoint (median of per-compound ratios T4/T3 = 2.27; abundance-weighted class-level T4/T1 = 5.29; Figure S4), suggesting frost-window accumulation across the variant-scaffold repertoire; given their structural heterogeneity, however, we restrict mechanistic interpretation to the four classical classes and treat the variant group as supporting, inventory-level evidence.

### 3.5. Senescence-Associated Regulation Extends Beyond the Source-Sink Model

The classical source-sink model attributes declining metabolite pools in senescing leaves to phloem export to sinks [60,61]. Our senescence-degradation hypothesis complements rather than replaces this view. Before T3, accumulation of PPT- and OT-type saponins reflects net biosynthetic gain while execution has not yet begun; between T3 and T4, the PPD decline likely combines degradation (vacuolar collapse, glycosidase exposure, aglycone oxidation) with export of deglycosylated intermediates. Disentangling these fluxes will require isotope tracing (e.g., ^13^C-labeled PPD precursors) and compartment-resolved metabolite analysis. Spatiotemporal metabolomic studies in *Arabidopsis* have similarly shown that senescence rewires metabolism in a stage- and tissue-specific manner [40]. Several alternative explanations for the frost-endpoint PPD decline were also considered; these explanations are not mutually exclusive and may act in combination. First, direct cold injury at T4—the membrane damage, cell shrinkage and wall invagination documented histologically (Figure 6D)—could itself drive saponin loss or transformation independently of the senescence program, and the present design cannot separate senescence-coupled catabolism from acute frost damage. Second, a concentration artifact from tissue desiccation can be excluded, because quantification was performed on lyophilized leaf powder extracted at a fixed dry-mass-to-solvent ratio (10 mg/mL), so the decline cannot reflect simple water loss. Third, source-sink remobilization is not mutually exclusive with degradation: phloem export of saponins or of deglycosylated intermediates toward the root would act in the same direction and is plausible in a perennial that resorbs nutrients before leaf shedding [59,60,61]. Fourth, oxidative degradation of exposed aglycones and dilution by residual biomass changes at T4 cannot be fully excluded with the present design; resolving their relative contributions will require isotope tracing and per-leaf biomass accounting, as outlined above.

Beyond these mechanistic considerations, our findings add a physiological dimension to ginsenoside research. Ginsenosides have traditionally been investigated as pharmacologically active end-products and quality markers, yet emerging evidence indicates that they also participate in the physiological regulation of the plant itself: ginsenosides Rb1 and Re dose-dependently regulate adventitious root formation through a CLE45-WOX11 regulatory module [65], and exogenous ginsenoside Rd modulates seed germination, seedling growth and the GA/ABA/IAA hormone balance in a concentration-dependent manner [66]. Conversely, developmental programs themselves gate saponin biosynthesis—floral development in *Panax notoginseng* drives a stage-specific burst of Rb3 accumulation through coordinated hormonal fluxes and transcription factor activity [67]—and endogenous ginsenoside levels remain essentially unchanged across seed dehiscence, arguing against a role as dormancy-release switches [68]. The class-specific, senescence-coupled accumulation programs resolved here extend this picture to leaf senescence: ginsenoside secondary metabolism is not a static defensive reservoir but is actively remodeled by the senescence program and may in turn feedback into physiological processes such as nutrient remobilization, rhizosphere allelopathy and dormancy preparation in this perennial medicinal plant. Ginsenosides should therefore be viewed as metabolites embedded in the physiological activities of the ginseng life cycle—a perspective that links secondary metabolism research with whole-plant physiology and opens new questions for functional validation.

### 3.6. Limitations and Outlook

Several limitations qualify our conclusions. (i) Transcriptomes cover T1–T3 only; the frost-terminated T4 window—where the PPD decline occurs—lacks expression data. T4 was an ad hoc sampling: an acute cold snap (an approximately 10 °C temperature drop two days after the T3 sampling) wilted and desiccated the canopy, no transcriptome collection had been planned for this stage, and the frost-affected state of the T4 tissue should be borne in mind when interpreting the endpoint metabolite changes. (ii) Transcriptome replication is unbalanced across stages—progressive RNA degradation during senescence reduced the qualified libraries from six per stage to six, four and two for T1, T2 and T3, respectively—and T3, the onset of the execution phase, thus has only two replicates, limiting statistical power precisely for the terminal senescence window; differential expression was therefore assessed with Welch’s *t*-test under FDR control, and gene-level inferences are interpreted jointly with the metabolomic and histological data. T3-level differential-expression calls are therefore treated as indicative and, where they inform the mechanistic interpretation, are corroborated by the fully replicated metabolomic trajectories (n = 3 per stage across all four stages) and by the histological series. (iii) Metabolome and transcriptome are mismatched in temporal resolution (four vs. three stages), limiting fine-grained coupling. (iv) All metabolome-transcriptome links are statistical associations, not causal relationships; functional validation of WRKY and *NAC087* through overexpression or silencing is needed. (v) Subcellular localization of saponins was not measured, and UGT/β-glucosidase activities were not assayed enzymatically, so the vacuolar-collapse/deglycosylation mechanism, although supported by metabolomic stratification and β-glucosidase induction, remains inferential; mass spectrometry imaging could map saponin redistribution directly, and quantitative proteomics could test whether transcript-level changes in CYP450s, UGTs and transcription factors translate into protein-level activity, including post-translational regulation. To consolidate the transcriptomic trends with larger sample sizes, publicly available *Panax* leaf transcriptome resources could be mined as external validation sets, and targeted qPCR assays of the key BGLU, UGT and senescence-executor genes across T1–T4 would close the terminal-window gap without requiring full transcriptomes. Likewise, future work with full replicate-level transcriptomic data would enable mixed-effects modeling of the metabolite-transcript relationships across the originally intended paired design. Together with isotope tracing and compartment-resolved profiling, these experiments would convert the present associative framework into a causal model. (vi) Metabolite annotation is mostly at the class level: the majority of features lack authentic-standard confirmation, so under the Metabolomics Standards Initiative framework [48] the classical-class assignments are putative (level 2, supported by diagnostic fragmentation) and the variant-aglycone features are class-level (level 3) annotations; in addition, the retention of [M–H]- and [M+FA–H]- ion forms as independent features, although justified and sensitivity-tested (Section 4.2), may influence feature counts and class-level quantitative inference. (vii) Because T4 is a frost-terminated endpoint rather than a stage of the natural senescence progression, every T4-inclusive statement in this study describes an endpoint at which late-senescence and frost effects cannot be fully separated; the T1–T3-restricted trend analyses were adopted for this reason. Targeted qPCR validation of the key BGLU, UGT and senescence-executor genes across T1–T4 has not yet been performed and remains necessary to confirm the transcript-level trends.

## 4. Materials and Methods

### 4.1. Plant Material and Sampling Design

Three-year-old field-grown *P. ginseng* (cv. Damaya) plants were cultivated at a ginseng production base in Fusong County, Jilin Province, China (41° N, 127° E). After the red-fruit stage, leaves were sampled at four sampling stages defined by visible phenotypes: T1 (post-red-fruit, fully green leaves), T2 (early senescence, yellowing initiating at leaf tips), T3 (late senescence, ~50% of leaf area yellowed) and T4 (frost-terminated, wilted, fully desiccated leaves). For each stage, leaves from three biological replicates were collected, flash-frozen in liquid nitrogen and stored at −80 °C. Metabolomic profiling covered all four stages (n = 3 per stage); methanolic extraction tolerated the desiccated state of T4 tissue and did not require RNA-grade material, allowing the frost-terminated stage to be profiled metabolomically even though it was inaccessible to transcriptomics. The T4 sampling was an ad hoc addition: two days after the T3 sampling, an acute cold snap (an approximately 10 °C temperature drop) rapidly wilted and desiccated the canopy, and the T4 leaves were collected from this frost-terminated material (see the frost-injury histology in Figure 6D); transcriptome sampling had not been planned for T4, and its RNA was too degraded for sequencing. Accordingly, T4 is treated throughout this study as a frost-terminated endpoint rather than as a stage of the natural senescence progression: temporal trend inference (GEE) is restricted to T1–T3, and T4 enters the analyses only as a distinct endpoint compared with T3. Transcriptome sequencing covered T1–T3 but not T4, because RNA from T4 wilted leaves was too degraded to meet sequencing quality requirements. Six leaf samples per stage were originally collected for RNA-seq; however, progressive RNA degradation during senescence rendered two T2 and four T3 libraries unusable, leaving six, four and two qualified biological replicates for T1, T2 and T3, respectively. Additional leaves of each stage were fixed for paraffin sectioning and microscopic observation. Representative field plants and leaves at each sampling stage are shown in Figure 2.

### 4.2. Targeted Metabolomic Profiling

Saponin profiling was performed on a Vanquish UPLC system coupled to a Q Exactive Plus high-resolution mass spectrometer (Thermo Fisher Scientific, Waltham, MA, USA; Orbitrap; resolution 70,000 FWHM at *m*/*z* 200), following widely used LC-MS-based metabolomic workflows for saponin and metabolite profiling [4,42,45,69]. Separation used an Accucore C18 column (100 mm × 2.1 mm, 1.8 μm) with 0.1% formic acid in water (A) and acetonitrile (B) as mobile phases (5–95% B over 25 min; 0.3 mL/min; 35 °C). Electrospray ionization was operated in negative mode (−2.7 kV; scan range *m*/*z* 100–1500). Dexamethasone was spiked into every sample as an internal standard and was consistently detected across all 12 runs, confirming instrument stability throughout the sequence; it was used exclusively for signal correction and analytical quality control. Data were processed with Compound Discoverer 3.1 (minimum peak intensity 500,000 counts; retention-time alignment tolerance ±0.2 min). Saponin features were annotated following the pseudo-targeted characterization strategy established for ginsenosides on high-resolution LC-MS platforms [46,70]. The majority of features were annotated without authentic-standard confirmation; under the Metabolomics Standards Initiative identification-confidence framework [48], the annotations of the four classical classes are therefore putative (level 2), supported by diagnostic MS/MS fragmentation, whereas the variant-aglycone Other saponins are class-level (level 3) annotations for which the exact aglycone connectivity remains tentative. The four major aglycone classes (PPT, PPD, OT and OA) were distinguished by their diagnostic sapogenin product ions. The number and type of glycosidic residues were assigned from sequential neutral losses (glc 162.053, rha 146.058, pet 132.042 Da). Malonylated and acetylated derivatives were recognized from their characteristic neutral losses (malonyl 86.000/43.990 Da; acetyl 42.011 Da). Features whose MS/MS spectra did not match the diagnostic product ions of these four classes were grouped as variant-aglycone Other saponins. In total, 385 metabolite features were quantified and classified by aglycone into six groups: PPT-type (n = 139), PPD-type (n = 87), OT-type (n = 32), OA-type (n = 40), variant-aglycone Other saponins (n = 78) and non-saponin small molecules (n = 9). During feature curation, non-saponin small molecules detected under the same name at different retention times were merged by retaining the feature with the highest mean abundance, and the three features of the dexamethasone internal standard were removed; in total 25 features were removed, reducing the matrix from 410 to 385 features. The initial 410-feature matrix thus comprised the 376 curated saponins plus nine non-saponin small molecules, 22 redundant non-saponin chromatographic peaks and three internal-standard features; only the 376 curated saponins entered trend and statistical analyses. The internal standard therefore entered no statistical modeling, trajectory or trend analysis and contributes no data to any result or conclusion of this study. Relative abundance was expressed as normalized peak area (CRPA), scaled to T1 = 1 for trajectory analysis. All 376 saponin features—unmodified glycosides as well as malonylated and acetylated derivatives—are reported at compound level in Table S1; the nine non-saponin small molecules are listed in Table S2. All downstream temporal analyses were restricted to the 376 saponin features.

A single saponin can generate more than one ion species during electrospray ionization—most commonly the deprotonated molecule [M − H]^−^ and its formate adduct [M + FA − H]^−^ (\Δ *m*/*z* = 46.0054, formic acid derived from the mobile phase)—and the two species are quantified by Compound Discoverer as independent features. Within the 376 saponin features, 20 high-confidence ion-form pairs (identical MS/MS-based annotation, \Δ *m/z* = 46.005 ± 0.01, co-elution within 0.1 min and temporal-profile Pearson r ≥ 0.9) were identified, involving 40 features (10.6% of the saponin feature matrix). These ion forms were deliberately retained as independent features for three reasons. First, feature-level quantification is the established convention in LC-MS-based metabolomic workflows. All downstream analyses used here (GEE, K-means clustering and principal component analysis (PCA)) are feature-level methods, and their temporal inference is not biased by ion-form multiplicity. Second, ginsenosides exhibit dense near-isobaric isomerism. Annotation- and *m*/*z*-based automated adduct merging would therefore risk collapsing genuine isomers. Indeed, other identically annotated feature pairs with the same 46.0054-Da spacing showed uncorrelated or even opposed temporal trajectories, demonstrating that they are distinct compounds rather than alternative ion forms. Third, in a sensitivity analysis, each of the 20 high-confidence pairs was merged into its more abundant member. This merging shifted class-level mean T4/T1 ratios by less than 7% and changed none of the qualitative conclusions. The 376 profiled saponin entities are therefore reported and interpreted as saponin features (ion-level measurements), consistent with feature-based metabolomic practice [42,69]. We nevertheless acknowledge that ion-form multiplicity can influence feature counts and, potentially, quantitative inference; for this reason we restrict mechanistic interpretation to class-level trajectories, which the sensitivity analysis shows to be robust.

### 4.3. Paraffin Sectioning and Microscopy

Leaf samples were fixed in FAA, dehydrated through a graded ethanol series, embedded in paraffin, sectioned, stained, and observed under a light microscope at 400× magnification. Sections of healthy (T1), early-senescent (T2), late-senescent (T3–T4) and frost-injured leaves were compared to identify senescence-associated histological features. The frost-injured leaves were collected from the same field two days after the T3 sampling, following an acute cold snap (an approximately 10 °C temperature drop), and served as an acute necrotic-damage contrast.

### 4.4. Transcriptome Sequencing and Analysis

Total RNA was extracted with the RNAprep Pure Plant Kit (TIANGEN), libraries were prepared with the TruSeq RNA Library Prep Kit v2 (Illumina, Inc., San Diego, CA, USA), and paired-end 150 bp sequencing was performed on an Illumina NovaSeq 6000. The cDNA libraries were sequenced on an Illumina platform by Genedenovo Biotechnology Co., Ltd. (Guangzhou, China). Reads were quality-controlled with fastp [71], aligned to the *P. ginseng* reference genome [72,73] with STAR [74], and quantified with RSEM [75] as transcripts per million (TPM) values; a total of 71,686 transcripts were annotated against KEGG, GO, NR and Swiss-Prot. Libraries were constructed and sequenced for six (T1), four (T2) and two (T3) biological replicates. Because negative-binomial differential-expression frameworks for RNA-seq data such as DESeq2 [76] cannot estimate dispersion reliably with two qualified replicates at T3, differential expression between T3 and T1 was assessed with Welch’s *t*-test on log_2_(TPM + 1) values under Benjamini–Hochberg FDR control, and genes with |log_2_FC| > 1 (computed on mean TPM with a 0.001 pseudocount) and FDR < 0.05 were considered differentially expressed; all gene-level expression changes discussed below meet both criteria unless explicitly noted. Per-replicate raw counts will be deposited together with the sequencing reads (Data availability). Given the unbalanced replication (T3, n = 2), Welch–Satterthwaite degrees of freedom for the T3–T1 contrast are correspondingly low, so the |log_2_FC| > 1 effect-size filter serves as the primary gate and FDR < 0.05 as the confirmatory statistical gate. Gene-level inferences are interpreted jointly with the metabolomic and histological data. Transcription factor families were annotated using PlantTFDB 4.0 [77].

### 4.5. Temporal Trend Analysis with Generalized Estimating Equations

Temporal effects on each saponin feature were modeled with generalized estimating equations (GEE) [78], using sampling stage as the predictor, restricted to the natural senescence progression (T1–T3), and CRPA as the response, with a Gaussian family (identity link), an exchangeable working correlation structure with biological replicate as the clustering group, and Huber–White sandwich variance estimation. All temporal and statistical analyses were performed on the curated 376-saponin matrix (Section 4.2). Because leaves of the sampled stages were collected by destructive sampling of different plants, so replicate identity carries no longitudinal meaning; nevertheless, the three replicate series of each feature (the same replicate measured at T1, T2 and T3) were specified as nominal exchangeable clusters—a conservative choice under which the Huber–White sandwich standard errors remain valid even if the working correlation structure is misspecified, providing robustness to heteroscedasticity and non-normality:

g(E[Y_it_]) = β_0_ + β_1_ × Time_t_,

where Y_it_ is the CRPA of saponin feature i at stage t and β_1_ quantifies the temporal effect. P-values were adjusted within each aglycone class by the Benjamini–Hochberg false discovery rate (FDR). The frost-terminated stage T4 was analyzed separately as a distinct endpoint: for each saponin feature, the log_2_ fold change between T4 and T3 was computed and tested with Welch’s *t*-test, and *p*-values were adjusted within each aglycone class by the Benjamini–Hochberg false discovery rate (class-internal FDR control). Saponin features were additionally grouped by K-means clustering of their standardized temporal profiles (k = 2 selected by elbow and silhouette criteria; Figure S5), and GEE statistics were summarized per cluster. Analyses were performed in Python with statsmodels and SciPy.

### 4.6. Metabolome-Transcriptome Joint Analysis

For each of the four classical aglycone classes (PPT, PPD, OT and OA), the class-level mean abundance (stages T1–T3) was correlated with per-gene mean TPM values using Spearman correlation on a 9-point matrix (3 stages × 3 metabolomic replicates), in which independent information derives from the three stage pairs while the metabolomic replicate structure stabilizes the ranking; the resulting associations were used for direction classification and gene-set enrichment rather than as independent-observation inference; associations with |r| > 0.3 and FDR < 0.05 were retained as significant. This analysis is exploratory: the nine points derive from only three independent stages, and within-stage metabolomic replicates inflate the apparent sample size, so *p*-values and FDR are interpreted descriptively and conclusions draw only on direction-level patterns and family-level enrichment. The proportion of true null hypotheses was estimated per class with Storey’s π_0_ statistic (λ = 0.5), yielding π_0_ approximately 0.43 for the PPT, PPD and OT classes versus approximately 1.0 for OA, confirming a strongly non-uniform *p*-value distribution for the former (Figure S6). The variant-aglycone Other-saponin group was excluded from this class-level analysis because its high structural heterogeneity means that the class-averaged profile does not represent a unified chemical entity. The directionality (positive vs. negative) of associated genes was compared among classes. Transcription factor family enrichment within class-associated gene sets was tested by Fisher’s exact test, with odds ratio (OR) > 2 and FDR < 0.05 considered significant.

## 5. Conclusions

By integrating targeted metabolomics of 376 saponin features, GEE-based temporal modeling, paraffin-section histology and transcriptomics, this study establishes a temporal atlas of ginsenoside accumulation across ginseng leaf senescence and proposes a hypothesis-level regulatory logic. First, our targeted profiling detected 385 metabolite features in senescing ginseng leaves. Of these, 376 were saponins (139 PPT-, 87 PPD-, 32 OT-, 40 OA-type and 78 variant-aglycone Other saponins), and nine were non-saponin small molecules. The saponin inventory spans one to five sugar moieties, with a median of two to three per class. Glucose is the predominant sugar, followed by apiose/pentose and rhamnose. The inventory comprises 259 unmodified glycosides together with 81 acetylated and 47 malonylated derivatives, with categories not mutually exclusive (Table S2). The nine non-saponin small molecules were recorded in this detection inventory only and were excluded from all temporal and accumulation-pattern analyses. This leaf-resolved inventory covers the aglycone and glycosylation diversity reported across ginseng tissues, in which ginsenosides accumulate in roots, leaves, stems, flowers and fruits with tissue-specific profiles [1,5,6,47], and it establishes senescing leaves as a chemically rich resource. Natural senescence globally reshaped this metabolome: of the 376 saponin features, 326 (86.7%) showed significant temporal effects across the T1–T3 gradient, and the four classical aglycone classes followed divergent programs—sustained accumulation (PPT, OT and PPD) versus a U-shaped curve (OA)—while variant-aglycone Other saponins accumulated progressively; at the frost-terminated endpoint T4, PPD-type saponins declined by 37% to the T1 level (abundance-weighted class-level T4/T3 = 0.63), whereas the other classes rose further. Second, the frost-endpoint PPD decline is glycosylation-dependent (per-compound median T4/T3 = 0.51 for low-glycosylation compounds vs. 1.04 for highly glycosylated ones) and is temporally coupled to the senescence execution phase marked by *SAG12* and *ENDO1* induction and histologically verified vacuolar collapse, consistent with a hypothesized vacuolar-collapse/glycosidase-exposure degradation mechanism that remains to be tested at the enzyme-activity and protein levels. Third, senescence signaling in ginseng leaves is organized around differential hormone responses (SA activation, JA and ethylene attenuation), WRKY/NAC-centered transcriptional control, and a dichotomous saponin–gene correlation pattern, with WRKY factors enriched (OR 3.61–4.04) among saponin-associated gene sets. Given the two-replicate T3 transcriptome, these signaling inferences are exploratory and require confirmation with larger replicate numbers. These findings provide the first integrated picture of how leaf senescence reshapes ginsenoside accumulation in a medicinal plant, with WRKY and *NAC087* nominated as candidate regulators for functional validation. Furthermore, targeted qPCR validation of BGLU, UGT and senescence-executor genes across T1–T4 is underway to close the T3–T4 expression gap.

## Figures and Tables

**Table 1:** Summary of generalized estimating equations (GEE) temporal-effect analysis by saponin group (GEE restricted to the T1–T3 natural senescence gradient; T4 analyzed separately as a frost endpoint).

Class	Features	Converged	Significant	Sig. Rate (%)	Up	Down	Mean Coefficient ± SD	Median Coefficient [IQR]
PPT	139	139	114	82.0	79	35	162,453 ± 1,003,732	7684 [−1732, 68,190]
PPD	87	87	77	88.5	56	21	779,751 ± 3,321,639	27,614 [−2696, 235,297]
OT	32	32	29	90.6	17	12	41,505 ± 130,400	2409 [−11,914, 40,060]
OA	40	39	37	92.5	23	14	−22,243 ± 128,344	3315 [−4515, 14,229]
Other saponins	78	77	69	88.5	49	20	29,938 ± 146,898	1373 [−1789, 12,895]
Total	376	374	326	86.7	224	102	–	–

Note. Significance rate uses the total number of compounds per class as denominator (FDR < 0.05). Up/Down counts significant compounds by the sign of the GEE temporal coefficient. Mean coefficient ± SD and median coefficient with interquartile range (IQR) are given in model units; the large SD-to-mean ratios reflect heavy right tails, so the median/IQR is the more robust summary.

**Table 2:** Summary of generalized estimating equations (GEE) temporal-effect analysis by K-means cluster.

Cluster	Features	Converged	Significant	Sig. Rate (%)	Up	Down	Mean Coefficient ± SD	Mean -Log_10_(p)
Cluster 0 (slow-rising)	231	231	200	86.6	127	73	6665 ± 30,465	79.53
Cluster 1 (steep-rising)	135	135	121	89.6	92	29	672,522 ± 2,831,662	133.36
Total	366	366	321	87.7	219	102	–	–

Note. Cluster assignments derive from K-means clustering (k = 2) of the standardized temporal profiles of 366 saponin features with complete standardized profiles in the cleaned data matrix. Cluster 0 = slow-rising group; Cluster 1 = steep-rising group; the mean temporal coefficient of Cluster 1 is 100.9-fold that of Cluster 0 (median ratio 58.9-fold; medians 91,650 vs. 1557), confirming that the difference is not driven by extreme values.

**Table 3:** Expression changes in senescence-related gene categories (T3 vs. T1).

Gene Category	Genes	Up	Down	Down (%)	Median Log_2_FC
Metacaspases	18	3	2	11.1	0.00
VPE	1	0	1	100.0	−2.24
Autophagy (ATG)	370	44	146	39.5	−0.61
Senescence-associated (SAG)	268	26	128	47.8	−0.91
Ethylene pathway	595	87	237	39.8	−0.47

Note. Up/Down defined by log_2_FC > 1/<−1 with a 0.001 pseudocount (Welch’s *t*-test on six, four and two biological replicates for T1, T2 and T3; see Section 4.4). Median log_2_FC is computed over all genes in the category.

## Data Availability

All processed data supporting the findings are included in the article and its Supplementary Tables. The raw transcriptome sequencing reads (24 paired-end FASTQ files from the 12 qualified leaf libraries) have been deposited in the Genome Sequence Archive (GSA) at the National Genomics Data Center (NGDC), China National Center for Bioinformation, under BioProject accession PRJCA076147 (BioSample accessions SAMC8724102-SAMC8724113; GSA accession CRA051315), and the raw LC-MS data (12 negative-ion RAW files) have been deposited in the NGDC OMIX archive under accession OMIX021567 (associated with the same BioProject). Both datasets are scheduled for public release on 1 February 2027 and will be released earlier upon publication.

## Supplementary Materials

The following supporting information can be downloaded at https://www.mdpi.com/article/10.3390/plants15203209/s1: Figure S1: Monotonic accumulation of protopanaxatriol (PPT)-type saponins; Figure S2: Monotonic accumulation of ocotillol (OT)-type saponins; Figure S3: U-shaped accumulation of oleanolic acid (OA)-type saponins; Figure S4: Plateau-then-surge accumulation of variant-aglycone Other saponins; Figure S5: Selection of K-means cluster number; Figure S6: Distributions of replicate-level Spearman correlation coefficients; Table S1: Compound-level data for all saponin features; Table S2: Saponin inventory and detection summary; Table S3: Stage-restricted saponin detection sets; Table S4: Feature-level generalized estimating equations (GEE) statistics; Table S5: Glycosylation-stratified permutation test and class-level summary of temporal and frost-endpoint statistics; Table S6: Expression statistics of the 123 annotated β-glucosidase genes.

## Abbreviations

The following abbreviations are used in this manuscript:

Ac	acetyl
ANOVA	analysis of variance
ARF	auxin response factor
ATG	autophagy-related
BGLU	β-glucosidase
bHLH	basic helix-loop-helix
CaM	calmodulin
CBL	calcineurin B-like
CIPK	CBL-interacting protein kinase
CPK	calcium-dependent protein kinase
CRPA	normalized peak area
CYP	cytochrome P450
ERF	ethylene response factor
FA	formic acid
FAA	formalin-acetic acid-alcohol
FDR	false discovery rate
GEE	generalized estimating equation
glc	glucose
gluA	glucuronic acid
GO	Gene Ontology
HD-ZIP	homeodomain-leucine zipper
IQR	interquartile range
JA	jasmonate
KEGG	Kyoto Encyclopedia of Genes and Genomes
LOX	lipoxygenase
Mal	malonyl
MAPK	mitogen-activated protein kinase
MEP	methylerythritol phosphate
MKK	MAPK
MVA	mevalonate
OA	oleanolic acid
OR	odds ratio
OT	ocotillol
PCA	principal component analysis
PCD	programmed cell death
pet	pentose
PPD	protopanaxadiol
PPT	protopanaxatriol
PR	pathogenesis-related
rha	rhamnose
ROS	reactive oxygen species
RT	retention time
SA	salicylic acid
SAG	senescence-associated gene
SEM	standard error of the mean
TF	transcription factor
TPM	transcripts per million
UGT	UDP-glycosyltransferase
UPLC	ultra-performance liquid chromatography
VPE	vacuolar processing enzyme

## References

[1] Christensen L.P. (2009). Ginsenosides chemistry, biosynthesis, analysis, and potential health effects. Adv. Food Nutr. Res..

[2] Ratan Z.A., Haidere M.F., Hong Y.H., Park S.H., Lee J.-O., Lee J., Cho J.Y. (2021). Pharmacological potential of ginseng and its major component ginsenosides. J. Ginseng. Res..

[3] Augustin J.M., Kuzina V., Andersen S.B., Bak S. (2011). Molecular activities, biosynthesis and evolution of triterpenoid saponins. Phytochemistry.

[4] Yang W.Z., Ye M., Qiao X., Liu C.-F., Miao W.-J., Bo T., Tao H.-Y., Guo D.-A. (2012). A strategy for efficient discovery of new natural compounds by integrating orthogonal column chromatography and liquid chromatography/mass spectrometry analysis: Its application in *Panax ginseng*, *Panax quinquefolium* and *Panax notoginseng* to characterize 437 potential new ginsenosides. Anal. Chim. Acta.

[5] Yang W.Z., Hu Y., Wu W.Y., Ye M., Guo D.A. (2014). Saponins in the genus *Panax* L. (Araliaceae): A systematic review of their chemical diversity. Phytochemistry.

[6] Yang W., Qiao X., Li K., Fan J., Bo T., Guo D.A., Ye M. (2016). Identification and differentiation of *Panax ginseng*, *Panax quinquefolium*, and *Panax notoginseng* by monitoring multiple diagnostic chemical markers. Acta Pharm. Sin. B.

[7] Han J.Y., Kim H.J., Kwon Y.S., Choi Y.E. (2011). The Cyt P450 enzyme CYP716A47 catalyzes the formation of protopanaxadiol from dammarenediol-II during ginsenoside biosynthesis in *Panax ginseng*. Plant Cell Physiol..

[8] Han J.Y., Hwang H.S., Choi S.W., Kim H.J., Choi Y.E. (2012). Cytochrome P450 CYP716A53v2 catalyzes the formation of protopanaxatriol from protopanaxadiol during ginsenoside biosynthesis in *Panax ginseng*. Plant Cell Physiol..

[9] Jung S.C., Kim W., Park S.C., Jeong J., Park M.K., Lim S., Lee Y., Im W.-T., Lee J.H., Choi G. (2014). Two ginseng UDP-glycosyltransferases synthesize ginsenosides Rg3 and Rd. Plant Cell Physiol..

[10] Wei W., Wang P., Wei Y., Liu Q., Yang C., Zhao G., Yue J., Yan X., Zhou Z. (2015). Characterization of *Panax ginseng* UDP-glycosyltransferases catalyzing protopanaxatriol and biosyntheses of bioactive ginsenosides F1 and Rh1 in metabolically engineered yeasts. Mol. Plant.

[11] Lu C., Zhao S., Wei G., Zhao H., Qu Q. (2017). Functional regulation of ginsenoside biosynthesis by RNA interferences of a UDP-glycosyltransferase gene in *Panax ginseng* and *Panax quinquefolius*. Plant Physiol. Biochem..

[12] Rahimi S., Kim J., Mijakovic I., Jung K.-H., Choi G., Kim S.-C., Kim Y.-J. (2019). Triterpenoid-biosynthetic UDP-glycosyltransferases from plants. Biotechnol. Adv..

[13] Qiu H., Su L., Wang H., Zhang Z. (2021). Chitosan elicitation of saponin accumulation in *Psammosilene tunicoides* hairy roots by modulating antioxidant activity, nitric oxide production and differential gene expression. Plant Physiol. Biochem..

[14] Hou L., Li S., Zhang F., Gu Y., Li J. (2022). Effect of exogenous jasmonic acid on physiology and steroidal saponin accumulation in *Dioscorea zingiberensis*. Plant Physiol. Biochem..

[15] Ma L.J., Liu X., Guo L., Luo Y., Zhang B., Cui X., Yang K., Cai J., Liu F., Ma N. (2024). Discovery of plant chemical defense mediated by a two-component system involving β-glucosidase in *Panax* species. Nat. Commun..

[16] Ma L.J., Liu J.Y., Fu W.Y., Bi H.-C., Yuan D., Wan J.-B. (2025). β-Glucosidase mediates ginsenoside hydrolysis in the leaves of *Panax* species. Ind. Crops Prod..

[17] Lim P.O., Kim H.J., Nam H.G. (2007). Leaf senescence. Annu. Rev. Plant Biol..

[18] Woo H.R., Kim H.J., Lim P.O., Nam H.G. (2019). Leaf senescence: Systems and dynamics aspects. Annu. Rev. Plant Biol..

[19] Guo Y., Ren G., Zhang K., Li Z., Miao Y., Guo H. (2021). Leaf senescence: Progression, regulation, and application. Mol. Hortic..

[20] Gan S., Amasino R.M. (1995). Inhibition of leaf senescence by autoregulated production of cytokinin. Science.

[21] Noh Y.S., Amasino R.M. (1999). Identification of a promoter region responsible for the senescence-specific expression of SAG12. Plant Mol. Biol..

[22] Perez-Amador M.A., Abler M.L., De Rocher E.J., Thompson D.M., van Hoof A., LeBrasseur N.D., Lers A., Green P.J. (2000). Identification of BFN1, a bifunctional nuclease induced during leaf and stem senescence in *Arabidopsis*. Plant Physiol..

[23] Hatsugai N., Kuroyanagi M., Yamada K., Meshi T., Tsuda S., Kondo M., Nishimura M., Hara-Nishimura I. (2004). A plant vacuolar protease, VPE, mediates virus-induced hypersensitive cell death. Science.

[24] Coll N.S., Vercammen D., Smidler A., Clover C., Van Breusegem F., Dangl J.L., Epple P. (2010). *Arabidopsis* type I metacaspases control cell death. Science.

[25] Minina E.A., Filonova L.H., Fukada K., Savenkov E.I., Gogvadze V., Clapham D., Sanchez-Vera V., Suarez M.F., Zhivotovsky B., Daniel G. (2013). Autophagy and metacaspase determine the mode of cell death in plants. J. Cell Biol..

[26] Yoshimoto K., Jikumaru Y., Kamiya Y., Kusano M., Consonni C., Panstruga R., Ohsumi Y., Shirasu K. (2009). Autophagy negatively regulates cell death by controlling NPR1-dependent salicylic acid signaling during senescence and the innate immune response in *Arabidopsis*. Plant Cell.

[27] van Doorn W.G. (2011). Classes of programmed cell death in plants, compared to those in animals. J. Exp. Bot..

[28] Kim J.H., Woo H.R., Kim J., Lim P.O., Lee I.C., Choi S.H., Hwang D., Nam H.G. (2009). Trifurcate feed-forward regulation of age-dependent cell death involving miR164 in *Arabidopsis*. Science.

[29] Li Z., Peng J., Wen X., Guo H. (2013). ETHYLENE-INSENSITIVE3 is a senescence-associated gene that accelerates age-dependent leaf senescence by directly repressing miR164 transcription in *Arabidopsis*. Plant Cell.

[30] Qiu K., Li Z., Yang Z., Chen J., Wu S., Zhu X., Gao S., Gao J., Ren G., Kuai B. (2015). EIN3 and ORE1 accelerate degreening during ethylene-mediated leaf senescence by directly activating chlorophyll catabolic genes in *Arabidopsis*. PLoS Genet..

[31] Cao H., Glazebrook J., Clarke J.D., Volko S., Dong X. (1997). The *Arabidopsis* NPR1 gene that controls systemic acquired resistance encodes a novel protein containing ankyrin repeats. Cell.

[32] Morris K., MacKerness S.A.H., Page T., John C.F., Murphy A., Carr J., Buchanan-Wollaston V. (2000). Salicylic acid has a role in regulating gene expression during leaf senescence. Plant J..

[33] Lorenzo O., Chico J.M., Sanchez-Serrano J.J., Solano R. (2004). JASMONATE-INSENSITIVE1 encodes a MYC transcription factor essential to discriminate between different jasmonate-regulated defense responses in *Arabidopsis*. Plant Cell.

[34] Miao Y., Zentgraf U. (2007). The antagonist function of *Arabidopsis* WRKY53 and ESR/ESP in leaf senescence is modulated by the jasmonic and salicylic acid equilibrium. Plant Cell.

[35] Zhou C., Cai Z., Guo Y., Gan S. (2009). An *Arabidopsis* mitogen-activated protein kinase cascade, MKK9-MPK6, plays a role in leaf senescence. Plant Physiol..

[36] Miao Y., Laun T., Zimmermann P., Zentgraf U. (2004). Targets of the WRKY53 transcription factor and its role during leaf senescence in *Arabidopsis*. Plant Mol. Biol..

[37] Guo Y., Gan S. (2006). AtNAP, a NAC family transcription factor, has an important role in leaf senescence. Plant J..

[38] Rushton P.J., Somssich I.E., Ringler P., Shen Q.J. (2010). WRKY transcription factors. Trends Plant Sci..

[39] Guo P., Li Z., Huang P., Li B., Fang S., Chu J., Guo H. (2017). A tripartite amplification loop involving the transcription factor WRKY75, salicylic acid, and reactive oxygen species accelerates leaf senescence. Plant Cell.

[40] Watanabe M., Balazadeh S., Tohge T., Erban A., Giavalisco P., Kopka J., Mueller-Roeber B., Fernie A.R., Hoefgen R. (2013). Comprehensive dissection of spatiotemporal metabolic shifts in primary, secondary, and lipid metabolism during developmental senescence in *Arabidopsis*. Plant Physiol..

[41] Li Y.Q., Kong D.X., Fu Y., Sussman M.R., Wu H. (2020). The effect of developmental and environmental factors on secondary metabolites in medicinal plants. Plant Physiol. Biochem..

[42] Liu J., Liu Y., Wang Y., Abozeid A., Zu Y.-G., Tang Z.-H. (2017). The integration of GC-MS and LC-MS to assay the metabolomics profiling in *Panax ginseng* and *Panax quinquefolius* reveals a tissue- and species-specific connectivity of primary metabolites and ginsenosides accumulation. J. Pharm. Biomed. Anal..

[43] Li J., Ma L., Zhang S., Zuo C., Song N., Zhu S., Wu J. (2019). Transcriptome analysis of 1- and 3-year-old *Panax notoginseng* roots and functional characterization of saponin biosynthetic genes DS and CYP716A47-like. Planta.

[44] Fan R., Zhu C., Qiu D., Mao G., Mueller-Roeber B., Zeng J. (2023). Integrated transcriptomic and metabolomic analyses reveal key genes controlling flavonoid biosynthesis in Citrus grandis ‘Tomentosa’ fruits. Plant Physiol. Biochem..

[45] Yao C.L., Pan H.Q., Wang H., Yao S., Yang W.-Z., Hou J.-J., Jin Q.-H., Wu W.-Y., Guo D.-A. (2018). Global profiling combined with predicted metabolites screening: Discovery of ginsenosides in the leaves of *Panax notoginseng* as a case study. J. Chromatogr. A.

[46] Jia L., Wang H.D., Xu X.Y., Wang H.M., Li X., Hu Y., Chen B.X., Liu M.Y., Gao X.M., Li H.F. (2022). An off-line three-dimensional liquid chromatography/Q-Orbitrap mass spectrometry approach enabling the discovery of 1561 potentially unknown ginsenosides from the flower buds of *Panax ginseng*, *Panax quinquefolius* and *Panax notoginseng*. J. Chromatogr. A.

[47] Yang W.Z., Bo T., Ji S., Qiao X., Guo D.A., Ye M. (2013). Rapid chemical profiling of saponins in the flower buds of *Panax notoginseng* by integrating MCI gel column chromatography and liquid chromatography/mass spectrometry analysis. Food Chem..

[48] Sumner L.W., Amberg A., Barrett D., Beale M.H., Beger R., Daykin C.A., Fan T.W.-M., Fiehn O., Goodacre R., Griffin J.L. (2007). Proposed minimum reporting standards for chemical analysis: Chemical Analysis Working Group (CAWG) Metabolomics Standards Initiative (MSI). Metabolomics.

[49] Woo H.R., Koo H.J., Kim J., Jeong H., Yang J.O., Lee I.H., Jun J.H., Choi S.H., Park S.J., Kang B. (2016). Programming of plant leaf senescence with temporal and inter-organellar coordination of transcriptome in *Arabidopsis*. Plant Physiol..

[50] Dai C., Lee Y., Lee I.C., Nam H.G., Kwak J.M. (2018). Calmodulin 1 Regulates Senescence and ABA Response in *Arabidopsis*. Front. Plant Sci..

[51] Ren H., Zhao X., Li W., Hussain J., Qi G., Liu S. (2021). Calcium signaling in plant programmed cell death. Cells.

[52] Berkey R., Bendigeri D., Xiao S. (2012). Sphingolipids and plant defense/disease: The “death” connection and beyond. Front. Plant Sci..

[53] Huysmans M., Buono R.A., Skorzinski N., Radio M.C., De Winter F., Parizot B., Mertens J., Karimi M., Fendrych M., Nowack M.K. (2018). NAC transcription factors ANAC087 and ANAC046 control distinct aspects of programmed cell death in the *Arabidopsis* columella and lateral root cap. Plant Cell.

[54] Jochum G.M., Mudge K.W., Thomas R.B. (2007). Elevated temperatures increase leaf senescence and root secondary metabolite concentrations in the understory herb *Panax quinquefolius* (Araliaceae). Am. J. Bot..

[55] Li T.S.C., Wardle D. (2002). Seasonal fluctuations of leaf and root weight and ginsenoside contents of 2-, 3-, and 4-year-old American ginseng plants. HortTechnology.

[56] Jackson C.J.C., Dini J.P., Lavandier C., Faulkner H., Rupasinghe H.P.V., Proctor J.T.A. (2003). Ginsenoside content of North American ginseng (*Panax quinquefolius* L. Araliaceae) in relation to plant development and growing locations. J. Ginseng. Res..

[57] Lommen W.J., Schenk E., Bouwmeester H.J., Verstappen F.W. (2006). Trichome dynamics and artemisinin accumulation during development and senescence of *Artemisia annua* leaves. Planta Med..

[58] Wang D., Zhu H., Chen K., Xu M., Zhang Y., Yang C. (2011). Saponin accumulation in the seedling root of *Panax notoginseng*. Chin. Med..

[59] Aerts R. (1996). Nutrient resorption from senescing leaves of perennials: Are there general patterns?. J. Ecol..

[60] Hortensteiner S., Feller U. (2002). Nitrogen metabolism and remobilization during senescence. J. Exp. Bot..

[61] Distelfeld A., Avni R., Fischer A.M. (2014). Senescence, nutrient remobilization, and yield in wheat and barley. J. Exp. Bot..

[62] Tansakul P., Shibuya M., Kushiro T., Ebizuka Y. (2006). Dammarenediol-II synthase, the first dedicated enzyme for ginsenoside biosynthesis, in *Panax ginseng*. FEBS Lett..

[63] Yang Z., Li X., Yang L., Peng S., Song W., Lin Y., Xiang G., Li Y., Ye S., Ma C. (2023). Comparative genomics reveals the diversification of triterpenoid biosynthesis and origin of ocotillol-type triterpenes in *Panax*. Plant Commun..

[64] Ye X.W., Li C.S., Zhang H.X., Li Q., Cheng S.-Q., Wen J., Wang X., Ren H.-M., Xia L.-J., Wang X.-X. (2023). Saponins of ginseng products: A review of their transformation in processing. Front. Pharmacol..

[65] Liu J., Chen T., Zhang J., Li C., Xu Y., Zheng H., Zhou J., Zha L., Jiang C., Jin Y. (2020). Ginsenosides regulate adventitious root formation in *Panax ginseng* via a CLE45-WOX11 regulatory module. J. Exp. Bot..

[66] Shen Y.L., Fan M.L., Li G., Li Q., Meng X.R., Wang E.P., Chen C.B. (2023). Allelopathic effect of ginsenoside Rd on ginseng seeds and seedlings. Appl. Ecol. Environ. Res..

[67] Wang C., Sun H., Yang Y., Peng C., Liu Y., Tao Y. (2025). Coordinated gene expression and hormonal fluxes dictating ginsenoside Rb3 biosynthesis in floral development of *Panax notoginseng*. BMC Plant Biol..

[68] Min J.E., Long N.P., Hong J.Y., Kim S.J., Anh N.H., Wang D., Wang X., Park J.H., Kwon S.W., Lee S.J. (2022). The dehiscence process in *Panax ginseng* seeds and the stigmasterol biosynthesis pathway in terms of metabolomics. J. Ginseng. Res..

[69] Chen W., Gong L., Guo Z., Wang W., Zhang H., Liu X., Yu S., Xiong L., Luo J. (2013). A novel integrated method for large-scale detection, identification, and quantification of widely targeted metabolites: Application in the study of rice metabolomics. Mol. Plant.

[70] Zuo T., Zhang C., Li W., Wang H., Hu Y., Yang W., Jia L., Wang X., Gao X., Guo D. (2020). Offline two-dimensional liquid chromatography coupled with ion mobility-quadrupole time-of-flight mass spectrometry enabling four-dimensional separation and characterization of the multicomponents from white ginseng and red ginseng. J. Pharm. Anal..

[71] Chen S., Zhou Y., Chen Y., Gu J. (2018). fastp: An ultra-fast all-in-one FASTQ preprocessor. Bioinformatics.

[72] Xu J., Chu Y., Liao B., Xiao S., Yin Q., Bai R., Su H., Dong L., Li X., Qian J. (2017). *Panax ginseng* genome examination for ginsenoside biosynthesis. GigaScience.

[73] Kim N.H., Jayakodi M., Lee S.C., Choi B., Jang W., Lee J., Kim H.H., Waminal N.E., Lakshmanan M., van Nguyen B. (2018). Genome and evolution of the shade-requiring medicinal herb *Panax ginseng*. Plant Biotechnol. J..

[74] Dobin A., Davis C.A., Schlesinger F., Drenkow J., Zaleski C., Jha S., Batut P., Chaisson M., Gingeras T.R. (2013). STAR: Ultrafast universal RNA-seq aligner. Bioinformatics.

[75] Li B., Dewey C.N. (2011). RSEM: Accurate transcript quantification from RNA-seq data with or without a reference genome. BMC Bioinform..

[76] Love M.I., Huber W., Anders S. (2014). Moderated estimation of fold change and dispersion for RNA-seq data with DESeq2. Genome Biol..

[77] Jin J., Tian F., Yang D.C., Meng Y.-Q., Kong L., Luo J., Gao G. (2017). PlantTFDB 4.0: Toward a central hub for transcription factors and regulatory interactions in plants. Nucleic Acids Res..

[78] Liang K.Y., Zeger S.L. (1986). Longitudinal data analysis using generalized linear models. Biometrika.

